# Computational Identification of Inhibitors Using QSAR Approach Against Nipah Virus

**DOI:** 10.3389/fphar.2019.00071

**Published:** 2019-02-12

**Authors:** Akanksha Rajput, Archit Kumar, Manoj Kumar

**Affiliations:** Virology Discovery Unit and Bioinformatics Centre, Institute of Microbial Technology, Council of Scientific and Industrial Research, Chandigarh, India

**Keywords:** Nipah virus, outbreak, inhibitors, QSAR, database, prediction algorithm

## Abstract

Nipah virus (NiV) caused several outbreaks in Asian countries including the latest one from Kerala state of India. There is no drug available against NiV till now, despite its urgent requirement. In the current study, we have provided a computational one-stop solution for NiV inhibitors. We have developed the first “*anti-Nipah”* web resource, which comprising of a data repository, prediction method, and data visualization module. The database contains of 313 (181 unique) chemicals extracted from research articles and patents, which were tested for different strains of NiV isolated from various outbreaks. Moreover, the quantitative structure–activity relationship (QSAR) based regression predictors were developed using chemicals having half maximal inhibitory concentration (IC_50_). Predictive models were accomplished using support vector machine employing 10-fold cross validation technique. The overall predictor showed the Pearson's correlation coefficient of 0.82 on training/testing dataset. Likewise, it also performed equally well on the independent validation dataset. The robustness of the predictive model was confirmed by applicability domain (William's plot) and scatter plot between actual and predicted efficiencies. Further, the data visualization module from chemical clustering analysis displayed the diversity in the NiV inhibitors. Therefore, this web platform would be of immense help to the researchers working in developing effective inhibitors against NiV. The user-friendly web server is freely available on URL: http://bioinfo.imtech.res.in/manojk/antinipah/.

## Introduction

Nipah virus infection is an emerging zoonotic infectious disease caused by Nipah virus (NiV). It is one of the important public health concerns in the South East Asian Region. The NiV is a negative sense single stranded RNA virus, belongs to genus *Henipavirus* and is a member of *Paramyxoviridae* family (Wang et al., 2001). The first outbreak of NiV was reported form Malaysia during 1998–1999 and thereafter-yearly outbreaks have been reported from Bangladesh or India (http://www.searo.who.int/entity/emerging_diseases/links/nipah_virus_outbreaks_sear/en/). NiV is known to infect various hosts viz., bats, pig, dog, cat, horse, and humans whereas fruit bats (genus *Pteropus*) remain as the main reservoir. The transmission of the virus can occur through direct contact with the contaminants of the infected bats. However, the human-to-human transmission can also be seen within families and in health care workers (Chadha et al., 2006; Luby et al., 2009). The incubation period of the NiV ranges from 5 to 14 days (https://www.cdc.gov/vhf/nipah/pdf/factsheet.pdf). Main clinical presentation seen among the NiV infected individuals involves the symptoms like fever and headache followed by encephalitis. Besides neurological manifestations, respiratory involvement was also documented in up to 69% of patients affected in Bangladesh-Indian outbreaks (Ang et al., 2018). The NiV infection has been reported to be associated with significant morbidity and mortality. The varied degree of mortality has been reported in various outbreaks. As per the World Health Organization, an average case fatality rate of 75% was observed for NiV infection (http://www.searo.who.int/entity/emerging_diseases/links/nipah_virus_outbreaks_sear/en/). In May 2018, India has witnessed another outbreak of NiV, after 2007, where 19 individuals were affected with a reported mortality of 94% (17/19) (http://www.who.int/csr/don/07-august-2018-nipah-virus-india/en/).

Despite being highly pathogenic, no prophylactic or therapeutic intervention is available against NiV. The supportive treatment consists of anticonvulsants is the mainstay for NiV infected patients (Ang et al., 2018). The efficacy of Ribavirin has also been evaluated against NiV infection. During the Malaysian outbreak, Ribavirin treatment resulted in a reduction of mortality rate to 32% compared to 54% in the control group (Chong et al., 2001). However, the later studies have reported that the Ribavirin alone or in combination with Chloroquine was ineffective for the survival of NiV infected hamsters (Georges-Courbot et al., 2006; Freiberg et al., 2010). Till now, various studies have been performed on the live virus as well on reporter assay systems. Around 19 compounds have been identified with EC50 or IC50 of >1μM against live NiV (Aljofan et al., 2009a,b, 2010; Freiberg et al., 2010; Lo et al., 2014, 2017, 2018a). A recent study by Dawes et al. have shown the antiviral effect of Favipiravir against live NiV Bangladesh and Malaysian isolates with EC50 of 14.8 and 44.8 μM, respectively (Dawes et al., 2018). Moreover, their *in-vivo* studies on hamsters have shown 100% survival rates.

However, there is still not any therapeutic modality available for NiV infection and not a single drug is under clinical trial against NiV. Thus, there is a need for the identification of other putative compounds or drugs against NiV. But owing to the biosafety level 4 (BSL-4) pathogen, limited studies could be conducted on the live NiV. Therefore, a wide range of compounds remains unexplored. Thus, there is a need for a computational tool that can identify the unexplored putative inhibitor against NiV.

The quantitative structure–activity relationship (QSAR) based predictive models are used to correlate the relationship between chemical structure and biological activity of chemicals through various molecular descriptors (Wang et al., 2015). The QSAR models works on the hypothesis of “similar compounds have similar structures” (Zhang et al., 2017). The molecular descriptors obtained from chemical structures helps in the development of the prediction model for the identification of new drugs (Lo et al., 2018b). We have previously developed the antiviral prediction servers by using QSAR algorithms mainly for overall viruses (AVCpred), Human immunodeficiency virus (HIVprotI), flaviviruses (Anti-flavi) (Gupta et al., 2016; Qureshi et al., 2017, 2018; Rajput and Kumar, 2018). Moreover, our department has developed various other prediction servers using QSAR based approach for the prediction of drugs against drug resistant *Mycobacterium tuberculosis* (MDRIpred), the anticancer activity of molecules (CacerIN), inhibitory molecules against Epidermal growth factor receptor (ntEGFR) etc. (Singla et al., 2013; Chauhan et al., 2014; Singh et al., 2016). However, in the present study, we have collected and manually curated overall anti-nipah inhibitors available in the research article and patents in form of a database and developed the first quantitative structure-activity relation (QSAR) based prediction algorithm using support vector machine (SVM) learning for the identification of anti-NiV compounds along the data visualization modules.

## Methods

### Data Collection

The experimentally validated compounds with anti-nipah activity were collected from research articles and patents. We used Pubmed (178 articles) and Orbit Intelligence (76 patents) using the search terms “Nipah,” “antiviral” OR “inhibit^*^.” The chemical information was fetched from PubChem or Chemspider or drawn using Marvinsketch. The data, representing inhibitory concentration 50 (IC_50_), effective concentration 50 (EC_50_), percentage inhibition and viral titers against NiV was obtained from 17 PMIDs and 01 patent. From the overall 181 unique NiV inhibitors, we proceeded to develop prediction algorithm with 95 compounds having IC_50_ values. We used the inhibitors with IC_50_, because it is considered as a standard for calculating the inhibition efficiency of any inhibitor and used in developing various algorithms (Chauhan et al., 2014; Qureshi et al., 2018; Rajput and Kumar, 2018). All the anti-nipah inhibitors with IC_50_ were converted into negative logarithm of half maximal inhibitory concentration (pIC_50_ = –log_10_(IC_50_(M))) for developing the regression- based predictive models (Kalliokoski et al., 2013; Rajput and Kumar, 2018). Hence, a total of 95 non-redundant anti-nipah compounds were used in the successive steps of descriptor calculation and model development.

### Data Preparation

The simplified molecular-input line-entry system (SMILES) of anti-nipah compounds were converted to 3D-standard data format (3D-SDF) followed by energy minimization using command line obabel software (O'Boyle et al., 2011). Further, the 3D-SDF format was introduced to chemical descriptor calculation.

### Descriptor Calculation

The chemical descriptors were calculated using PaDel software (Yap, 2011). Initially, a total of 17967 descriptors were extracted, which includes 1D, 2D, 3D, and fingerprints. Further, these overall chemical descriptors were used for the feature selection.

### Feature Selection

The feature selection was performed to remove non-desirable descriptors using preprocess “RemoveUseless” followed by the attribute evaluator “CfsSubsetEval”(Hall et al., 2009). For the regression approach most relevant 42 features were extracted from a total of 17,967. The detail of the relevant features is given in Supplementary Table S1.

### Machine Learning

**Figure 1:**
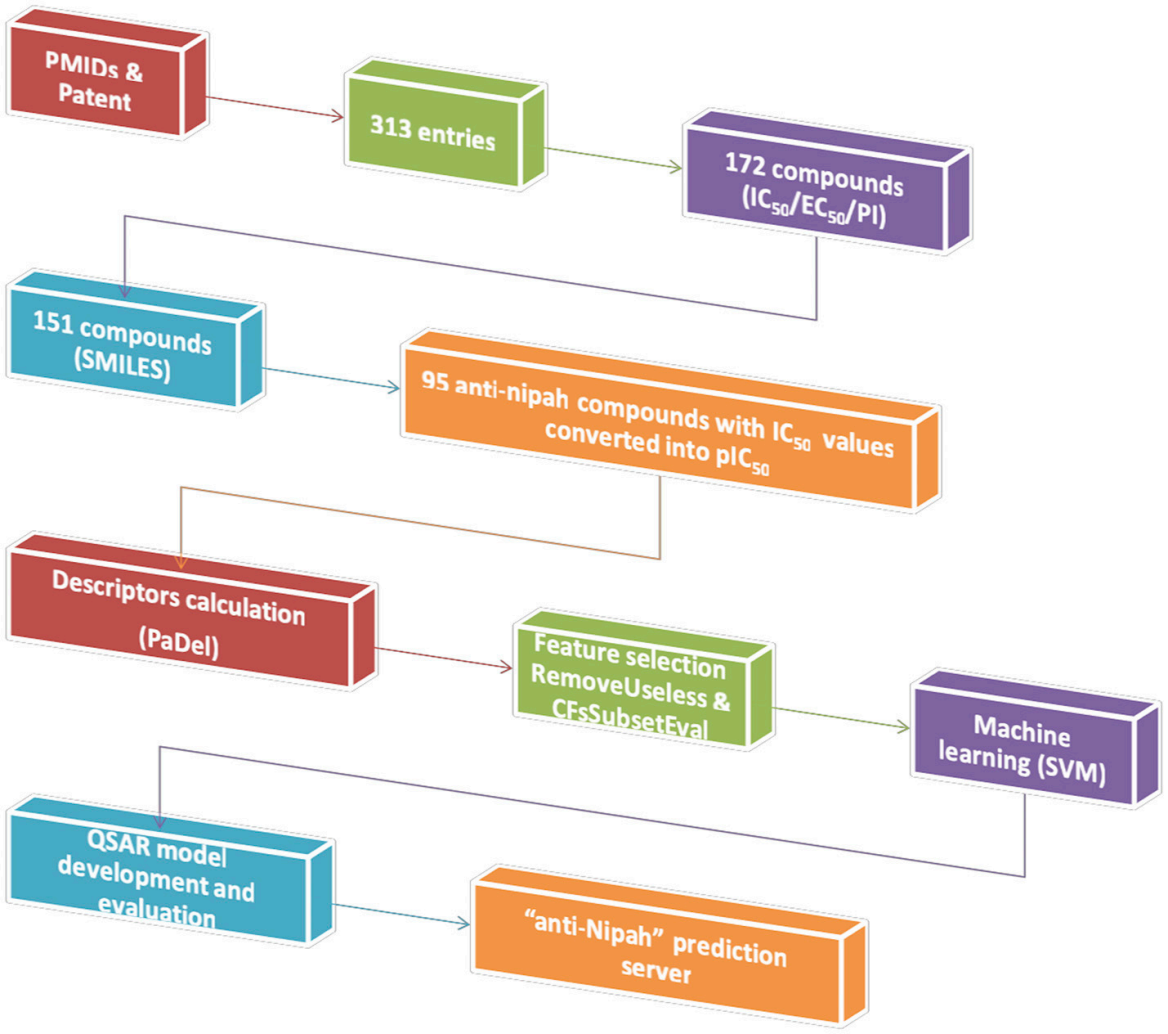
Overall architecture for the development of anti-Nipah prediction server.

The QSAR model was developed for anti-Nipah compounds using selected descriptors through support vector machine (Rajput et al., 2018). The architecture for the development of prediction model is given in Figure 1. We employed the 10-fold cross-validation approach for model development. The overall 95 dataset was divided into 85 training/testing and 10 independent validation data set using randomization method (Rajput et al., 2015). The robustness of the model was evaluated by performing internal as well as external cross-validation.

### Model Evaluation

The performance of the QSAR model was evaluated using Pearson's correlation coefficient (PCC,R), coefficient of determination (R^2^), mean absolute error (MAE), and root mean absolute error (RMSE), by using below stated formulas:

$$\begin{array}{l} R=\\ \frac{n\sum_{n=1}^{n}E_{i}^{act}E_{i}^{pred}-\sum_{n=1}^{n}E_{i}^{act}\sum_{n=1}^{n}E_{i}^{pred}}{\sqrt{n\sum_{n=1}^{n}{\left(E_{i}^{act}\right)}^{2}-{\left(\sum_{n=1}^{n}E_{i}^{act}\right)}^{2}}-\sqrt{n\sum_{n=1}^{n}{\left(E_{i}^{pred}\right)}^{2}-{\left(\sum_{n=1}^{n}E_{i}^{pred}\right)}^{2}}} \end{array}$$

$$\begin{array}{l} MAE=\frac{1}{n}\overset{n}{\underset{n=1}{\sum }}|E_{i}^{pred\;}-\;E_{i}^{act}| \end{array}$$

$$\begin{array}{l} RMSE=\sqrt{\frac{1}{n}\overset{n}{\underset{n=1}{\sum }}{\left(E_{i}^{pred\;}-\;E_{i}^{act}\right)}^{2}} \end{array}$$

where, n, $E_{i}^{pred}$, $E_{i}^{act}$ are size of dataset, predicted and actual efficiencies.

### Applicability Domain

The applicability domain was checked using William's plot and scatter plot. The William's plot showed the correlation between standardized residuals and leverage. The leverage (h) is related to Hotelling's t-squared statistics (*t*^2^) and Mahalanobis distance from centroid of the training/testing dataset. In general, the warning leverage threshold (h^*^) is set at 3^*^p/n, where p is number of selected descriptors plus one and n is number of compounds in training dataset. While the standard residuals cutoff was set at thrice the standard deviation ±3σ. Therefore, the predictive model considered to be reliable if maximum data points lies within the warning threshold of standard residuals and leverage (Tropsha et al., 2003).

We also checked the scatter plot between actual and predicted values of pIC50 along with checking the Spearman's correlation coefficient between them using R. The predicted models would be considered as robust if the data point concentrated near the trendline (Qureshi et al., 2018).

### Decoy Set

The robustness of the predictive model was also checked by decoy set using RApid DEcoy Retriever (RADER) software (Wang et al., 2017). The decoy set are the chemicals with similar H-bond donor, molecular weight, and logP but chemically dissimilar than active molcules (Rajput and Kumar, 2018).

### Chemical Clustering

The clustering of 95 compounds along with their IC_50_ was performed using “compounds specific bioactivity dendrogram” (C-SPADE), an online web based server (Ravikumar et al., 2017). The C-SPADE utilizes the structural information for the clustering of the compounds and which can be visualized as dendrogram along with their activity.

## Results

### Database

**Figure 2:**
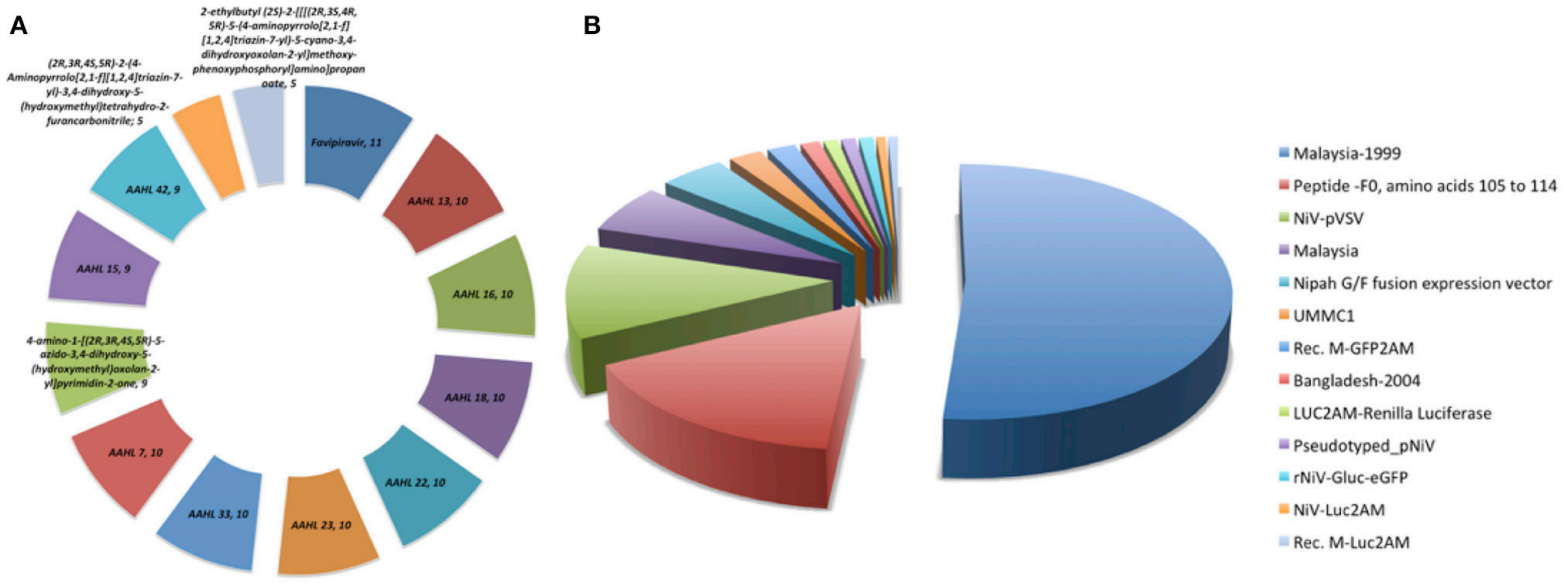
Frequency distribution of **(A)** anti-Nipah drugs, **(B)** Nipah virus strains targeted in the experiments.

The anti-Nipah database contains total 313 entries with 182 unique against Nipah virus with EC_50_, IC_50_, and percentage inhibition values. The database contains the fields like inhibitor name, NiV strain, experimental approaches, time and duration on inhibitor delivery, mode of delivery, type of inhibition, survival, and cytotoxicity activity, etc. The anti-Nipah compounds experimentally tested found majorly are Flavipiravir in 11 cases followed by AAHL 13, AAHL 16, AAHL 18, AAHL 22, AAHL 23, AAHL 33, AAHL 7 in 10 experiments each. The top-most acting drugs are shown in Figure 2A. Various NiV strains were targeted in developing the anti-Nipah compounds e.g., Malaysia-1999, NiV-pVSV, Malaysia, fusion expression vector in 160, 37, 19, and 14 experiments, respectively (Figure 2B).

Moreover, several assays are being used to check the inhibition of compounds against NiV. Maximally used assay is Chemiluminescent in 89 experiments followed by CatL-Peptide cleavage, Plaque assay, immunolabelling assay, Cytopathic effect assay in 50, 42, 41, and 22, entries respectively (Supplementary Figure S1).

### Prediction

#### Performance the QSAR Model

For the identification of the significant features of NiV the regression correlation test was performed between the chemical features and the pIC_50_ of NiV compounds using dataset of 95 compounds. Among 42 descriptors *viz, AATSC5e, MATS5e, JGI9, JGI10, FP169, FP204, FP339, FP396, FP490, FP551, FP582, FP606, ExtFP79, ExtFP442, ExtFP584, ExtFP700, ExtFP1010, ExtFP1019, GraphFP158, GraphFP504, GraphFP622, GraphFP762, GraphFP860, GraphFP906, GraphFP1007, MACCSFP26, MACCSFP150, SubFP147, KRFP349, KRFP360, KRFP364, KRFP397, KRFP607, KRFP1538, KRFP2135, KRFP3940, KRFPC349, KRFPC2135, KRFPC2694, KRFPC3139, KRFPC3520, KRFPC4292* were found most relevant for regression-based model development. During the 10-fold cross validation approach the training/testing and independent validation dataset displayed the PCC of 0.82 and 0.92, respectively. The values for all the statistical parameters are given in Table 1. Moreover, the actual and predicted pIC50 of anti-nipah compounds are shown in Supplementary Table S2. Apart from using independent validation dataset, the reliability of the training/testing model was cross-checked by decoy set. The prediction of decoy set showed that the model doesn't able to predict them with high accuracy as compared to anti-nipah compounds as shown in Supplementary Table S3.

**Table 1:** Performance of Support Vector Machine models on training/testing (85) and independent validation (10) data sets using 10-fold cross validation.

**pIC50**	**Descriptors**	**Pr-cor**	**cof-R2**	**RMSE**	**MAE**	**Parameters**
**RANDOM1**						
Training/Testing	42/17968	0.82	0.67	0.62	0.40	g = 0.005 c = 200
Independent validation	42/17968	0.85	0.64	0.66	0.58	g = 0.005 c = 200
**RANDOM2**						
Training/Testing	42/17968	0.81	0.65	0.65	0.50	g = 0.001 c = 500
Independent validation	42/17968	0.92	0.80	0.43	0.29	g = 0.001 c = 500
**RANDOM3**						
Training/Testing	42/17968	0.85	0.72	0.58	0.42	g = 0.001 c = 200
Independent validation	42/17968	0.79	0.59	0.68	0.57	g = 0.001 c = 200

*PCC, Pearson' correlation coefficient; cof-R^2^, coefficient of R^2^; RMSE, Root mean squatted error; MAE, Mean absolute error*.

#### Applicability Domain

**Figure 3:**
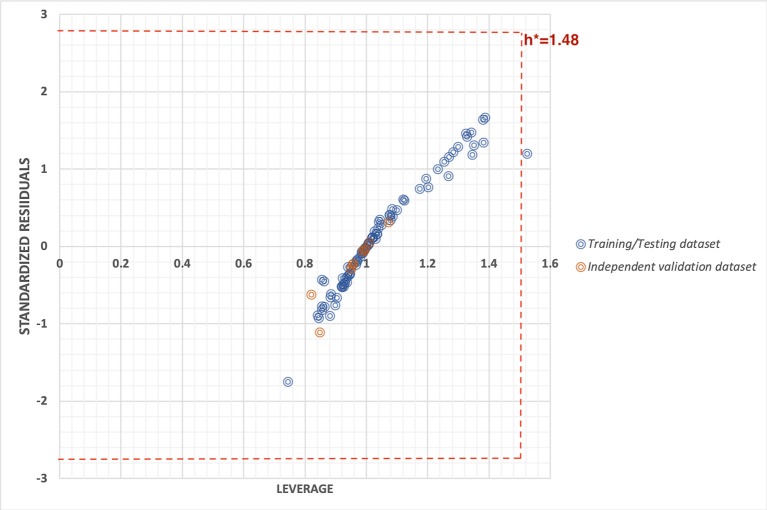
William's plot to check the applicability domain of training/testing and independent validation datasets plotted between standard residuals and leverage.

To check the robustness of the prediction model, we used two statistical approaches i.e., by plotting the William's plot against leverage and standard residuals along with the scatter plot between actual and predicted values. The William's plot showed the h^*^ of 1.48(=3p/n) while the cutoff standard deviation of ±2.78 (3σ). Almost all the data points lies within the threshold values of leverage and standard residuals (Figure 3).

**Figure 4:**
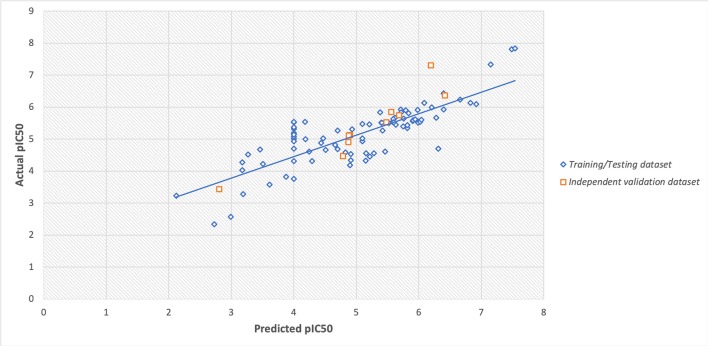
Scatter plot between actual and predicted values of pIC_50_ of training/testing and independent validation datasets.

However, the scatter plot between actual and predicted pIC50 displayed the maximum training/testing and independent validation dataset lies close to the trendline (i.e., x = y axis) with Spearman's correlation coefficient of 0.80. The scatter plot of actual v/s predicted values is provided in Figure 4.

#### Web Server

The QSAR based prediction model of anti-Nipah compounds or drugs was amalgamated into an openly available and easy to execute web server, “anti-Nipah.” The users can use anti-Nipah for the prediction of antiviral activity of the compounds or drugs against NiV. The “*anti-Nipah*” is freely available on url: http://bioinfo.imtech.res.in/manojk/antinipah/ and consisted of following features:

*Database browse and search:* User can get the information of available NiV inhibitors available in the literature as well as patents. The anti-Nipah browse can be performed on four important fields like NiV inhibitors, Strains, Assays, and mode of inhibitor delivery. Moreover, the user can check the information of respective compounds in the database through the search tool option of the server like viral strains, experimentation, assays performed, etc.

*Predictor (Input and Output):* The user can submit the query compound to the server. The server in return will predict the potency of the query compound against NiV. It will suggest the user for the potential antiviral activity of the query compound against NiV. Moreover, the user can also check the other drug-likeliness properties such as molecular formula, formal charges, H-bond acceptor and donor, Lipinski acceptor and donor, rotatable bonds, etc. in the server.

*Draw:* The Marvin sketch tool is incorporated into the “anti-Nipah” server so that user can also draw their query compounds. After submission of the structure, the user will know about the activity and chemical properties of the query compound.

#### Chemical Clustering

**Figure 5:**
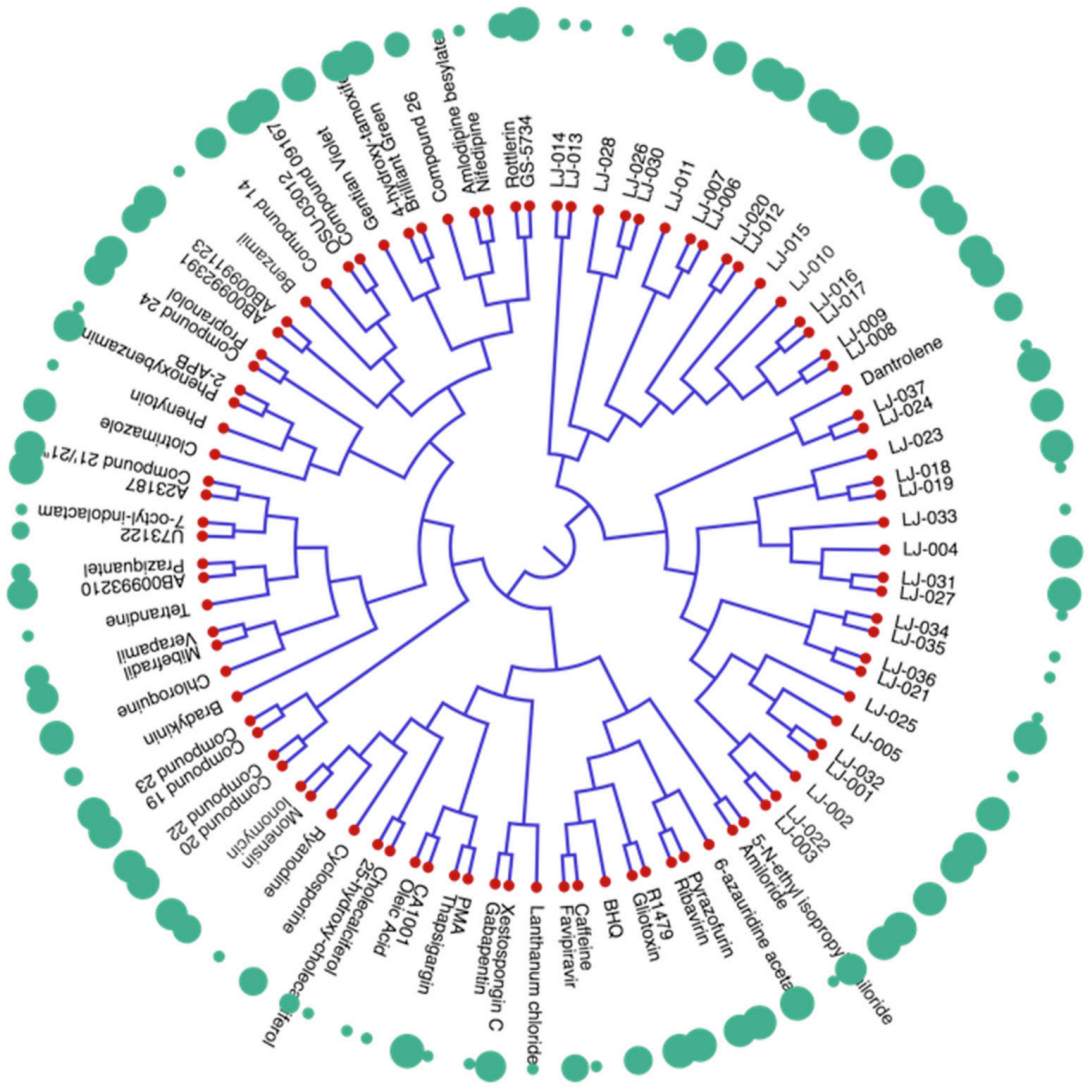
Dendrogram of inhibitors of Nipah virus (NiV): The red nodes represent the respective compound tested against NiV. The green spheres represent the activity of the compounds. The large spheres represent the compounds having high anti-Nipah activity while small spheres represent the less effective/inactive compounds.

The dendrogram of the 95 compounds were constructed as shown in Figure 5. The SMILES information was used for clustering using extended-connectivity fingerprint 4 (ECFP 4) module of the C-SPADE. Large shape spheres represent the active compounds (low IC_50_) while the less effective/inactive compounds (high IC_50_) can be seen as small spheres. Moreover, the active compounds were clustered together in the dendrogram.

## Discussion

The presence of antiviral prediction web servers is important for combating the emerging viral infections speedily. It become more important, especially for the viruses against which no treatment modality is available. Although, the limited antiviral prediction algorihtms are available for the prediction of compounds against viral infections, which includes the AVCpred, HIVprotI, and Anti-flavi (Qureshi et al., 2017, 2018; Rajput and Kumar, 2018). The AVCpred is an antiviral compound prediction server, especially for viruses HIV, HCV, HBV, HHV and also, include a general prediction tool for 26 viruses. While HIVprotI is a dedicated prediction server for compounds specifically targeting the integrase, protease and reverse transcriptase of HIV. The Anti-flavi web based predcition algorithm is helpful to predict and identify inhibitors against flaviviruses. However, in the present study, we have developed the first QSAR based “anti-Nipah” prediction web server for the screening of compounds having antiviral activity against NiV.

We have developed the first database (313 entries) with all the inhibitors of Nipah Virus, which include 120 chemical inhbitors which are highly diverse and varies in molecular weight from 182 to 1,000 g/mol. The maximally validated anti-nipah inhibitor is Flavipiravir, which is a purine analog and one amongst the effective broad-specturm antiviral effective against RNA viruses (Dawes et al., 2018). Followed by, several novel antivirals designed to target the replication stage of NiV (Aljofan et al., 2009a). Likewise, the user can get the details of all the NiV inhibitors available in reseach artciles as well as patents at a platform using our web resource. Further, using the experimentally validated data, we developed a robust QSAR based prediction method. This is the first QSAR based web server (integrating recursive regression model) dedicated to Nipah virus. The user can predict the anti-NiV activity of any unknown compound with the PCC of 0.82. Thus, our predictor would be very helpful to experimental biologists for speeding up their research toward developing novel and effective anti-NiV scaffolds.

The robustness of the prediction models were cross-checked using various statistical approaches like using independent validation dataset, applicability domain (William's plot), scatter plot, and decoy set. The independent validation dataset is considered as the external validation of the model, and we got a good PCC of 0.92, that showes that developed predictive model possess very high efficiency to predict an unknown molecule efficiently. Further, the applicability domain showed that the model is highly robust as majority of the datapoints lies within the cutoff threshold of leverage and standard residuals. Likewise, the scatter plot between actual and predicted accuracies further support the robustness of model with Spearman's and Pearson's correlation coefficient of 0.80 and 0.82, respectively. Moreover, the low predcition efficiency of decoy set further confirm the efficiency of prediction model. Thus, all the multivariate analysis approach proves the relaibilty of the model.

The similarity analyses showed the diversity of the anti-NiV compounds. Highly effective (low IC50) were clustered together, while the less effective/inactive (high IC50) compounds are scattered throughout the dendrogram. Thus, the presence of similar scaffold or chemical modifications might have resulted in the clustering of the active compounds. Interstingly, while performing the chemical clustering analysis, we found that in a few cases, highly efficient and less efficient compounds were also clustered together. The chemical modifications in functional groups might have increased their activity against NiV. Most of LJ series compounds, synthesized using side chain modification, were found to be clustered together. A modification in either carbon chain, carbon ring, or side group may have caused the transient shift in the activity of these compound. Therefore, the construction of chemical libraries using side group modifications can be useful for screening the effective antiviral compounds against NiV. Thus, our anti-Nipah prediction tool will be useful in the screening of such compounds against NiV. As NiV is a BSL-4 pathogen, therefore large scale screening for NiV inhibitors is a limiting factor. Hence, this becomes the limitation of our predictor as it is developed on the less number of chemicals available in literature. However, it is the first initiative toward developing the NiV dedicated computaional tool, but we will update our method as we get new updated data of NiV inhibitors.

## Conclusions

Nipah Virus is known for several outbreaks, with the recent one is from Kerala, India from May–June 2018. NiV is responsible for causing significant mortality, varies from 0 to 100%, among affected individuals. Therefore, in the current study we tried to investigate the anti-Nipah agents. Our first approach is to compile all the anti-Nipah agents available in PubMed and patents. Followed by developing the QSAR based regression predictor, and providing all the inhibitors in form of a user-friendly web interface named “anti-Nipah.” We hope that our web resource would prove beneficial for researchers to predict effective anti-nipah agents.

## Author Contributions

MK conceived the idea and helped in the interpretation, analysis, and overall supervision. AK collected data. AK and AR performed the curation. AR worked on the predictive models and web server development. AR, AK, and MK carried out the analysis. AK, AR, and MK wrote the manuscript.

### Conflict of Interest Statement

The authors declare that the research was conducted in the absence of any commercial or financial relationships that could be construed as a potential conflict of interest.
