# Supplementary material for: Computational Identification of Inhibitors Using QSAR Approach Against Nipah Virus
Source: Front Pharmacol. 2019 Feb 12;10:71. doi: 10.3389/fphar.2019.00071 (PMC6379726; doi:10.3389/fphar.2019.00071)
Supplement: Supplementary file 1 [file Data_Sheet_1.PDF]

Computational identification of inhibitors using QSAR approach against Nipah virus

Akanksha Rajput#, Archit Kumar# and Manoj Kumar *

Virology Discovery Unit and Bioinformatics Centre, Institute of Microbial Technology, Council of Scientific and Industrial Research (CSIR), Sector 39A, Chandigarh-160036, India.

# Equal contributions
* Corresponding Author: manojk@imtech.res.in

Supplementary Information

Supplementary Figures
Supplementary Figure S1. Frequency distribution of assays used to check the inhibition of anti-Nipah compounds

Supplementary Tables
Supplementary Table S1. Details of the most relevant descriptors extracted from RemoveUseless and CfsSubsetEval feature selection algorithms and used for prediction model development

Supplementary Table S2. Actual and predicted efficiency of 95 anti-nipah compounds employing support vector machine along with their structures

Supplementary Table S3. Details of prediction done using 74 decoy set through prediction model employing support vector machine along with their structures

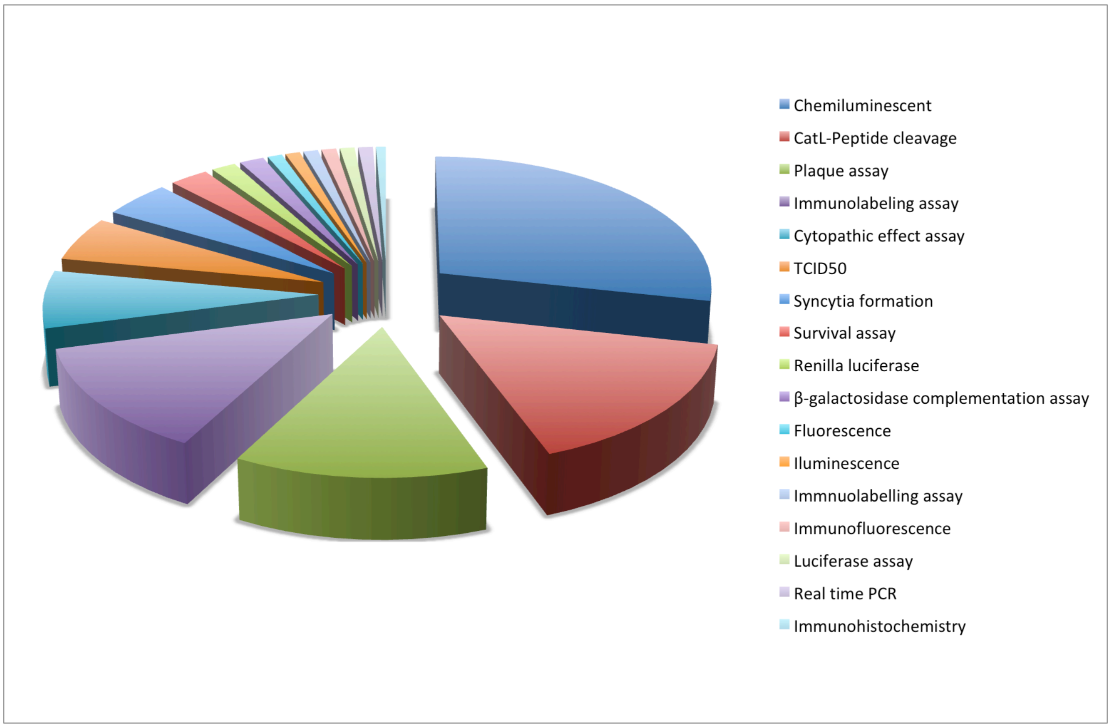

Supplementary Figure S1. Frequency distribution of assays used to check the inhibition of anti-Nipah compounds

Supplementary Table S1. Details of the most relevant descriptors extracted from RemoveUseless and CfsSubsetEval feature selection algorithms and used for prediction model development

Features	Description	Type
AATSC5e	Average centered Broto-Moreau autocorrelation - lag 5 / weighted by Sanderson electronegativities	2D
MATS5e	Moran autocorrelation - lag 5 / weighted by Sanderson electronegativities	2D
JGI9	Mean topological charge index of order 9	2D
JGI10	Mean topological charge index of order 10	2D
FP169	Fingerprint of length 1024 and search depth of 8	CDK fingerprint
FP204	Fingerprint of length 1024 and search depth of 8	CDK fingerprint
FP339	Fingerprint of length 1024 and search depth of 8	CDK fingerprint
FP396	Fingerprint of length 1024 and search depth of 8	CDK fingerprint
FP490	Fingerprint of length 1024 and search depth of 8	CDK fingerprint
FP551	Fingerprint of length 1024 and search depth of 8	CDK fingerprint
FP582	Fingerprint of length 1024 and search depth of 8	CDK fingerprint
FP606	Fingerprint of length 1024 and search depth of 8	CDK fingerprint
ExtFP79	Extends the Fingerprinter with additional bits describing ring features	CDK extended fingerprint
ExtFP442	Extends the Fingerprinter with additional bits describing ring features	CDK extended fingerprint
ExtFP584	Extends the Fingerprinter with additional bits describing ring features	CDK extended fingerprint
ExtFP700	Extends the Fingerprinter with additional bits describing ring features	CDK extended fingerprint
ExtFP1010	Extends the Fingerprinter with additional bits describing ring features	CDK extended fingerprint
ExtFP1019	Extends the Fingerprinter with additional bits describing ring features	CDK extended fingerprint
GraphFP158	Specialized version of the Fingerprinter which does not take bond orders into account	CDK graph only fingerprint
GraphFP504	Specialized version of the Fingerprinter which does not take bond orders into account	CDK graph only fingerprint
GraphFP622	Specialized version of the Fingerprinter which does not take bond orders into account	CDK graph only fingerprint
GraphFP762	Specialized version of the Fingerprinter which does not take bond orders into account	CDK graph only fingerprint
GraphFP860	Specialized version of the Fingerprinter which does not take bond orders into account	CDK graph only fingerprint
GraphFP906	Specialized version of the Fingerprinter which does not take bond orders into account	CDK graph only fingerprint
GraphFP1007	Specialized version of the Fingerprinter which does not take bond orders into account	CDK graph only fingerprint
MACCSFP26	MACCS keys	MACCS fingerprint
MACCSFP150	MACCS keys	MACCS

		fingerprint
SubFP147	Presence of SMARTS Patterns for Functional Group Classification by Christian Laggner	Substructure fingerprint
KRFP349	Presence of chemical substructures	Klekota-Roth fingerprint
KRFP360	Presence of chemical substructures	Klekota-Roth fingerprint
KRFP364	Presence of chemical substructures	Klekota-Roth fingerprint
KRFP397	Presence of chemical substructures	Klekota-Roth fingerprint
KRFP607	Presence of chemical substructures	Klekota-Roth fingerprint
KRFP1538	Presence of chemical substructures	Klekota-Roth fingerprint
KRFP2135	Presence of chemical substructures	Klekota-Roth fingerprint
KRFP3940	Presence of chemical substructures	Klekota-Roth fingerprint
KRFPC349	Count of chemical substructures	Klekota-Roth fingerprint count
KRFPC2135	Count of chemical substructures	Klekota-Roth fingerprint count
KRFPC2694	Count of chemical substructures	Klekota-Roth fingerprint count
KRFPC3139	Count of chemical substructures	Klekota-Roth fingerprint count
KRFPC3520	Count of chemical substructures	Klekota-Roth fingerprint count
KRFPC4292	Count of chemical substructures	Klekota-Roth fingerprint count

Supplementary Table S2. Actual and predicted efficiency of 95 anti-nipah compounds employing support vector machine along with their structures

S.No .	Anti_NiV _IDs	SMILES	Image	Actual_pIC50	Predicted_pIC50
1	Anti_NiV_	FC(F)(F)C(=O)NC1=C(C(=O)NC2=CC=CC3=CC=CC=C23)C2=C(CCCC2)S1	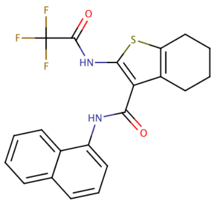	7.54	7.77
2	Anti_NiV _002	CCC(CC)COC(=O)C(C)NP(=O)(OCC1C(C(C(O1)(C#N)C2=CC=C3N2N=CN=C3N)O)O)OC4=CC=CC=C4	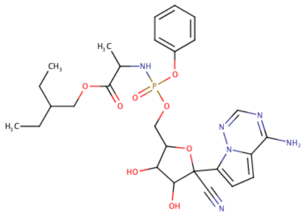	7.49	7.52
3	Anti_NiV_ 003	CCCCCCCC(=O)OC1C2C(=C(C1OC(=O)C(=CC)C)C)C3C(C(CC2(C)OC(=O)C)OC(=O)CCC)(C(C(=O)O3)(C)O)O	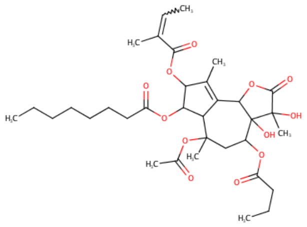	7.30	6.04
4	Anti_NiV_ 004	CC1CCC2(C(CC(C(O2)C(C)C(=O)C3=CC=CN3)C)C)OC1CC4=NC5=C(O4)C=CC(=C5C(=O)O)NC	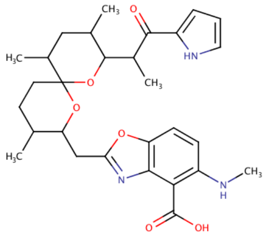	7.15	7.00
5	Anti_NiV_ 005	CC(=O)OCC1C(C(C(O1)N2C(=O)NC(=O)C=N2)O)O	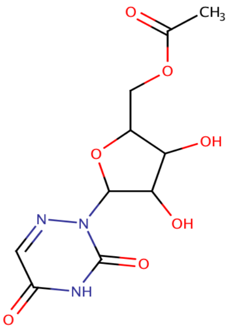	6.92	6.23
6	Anti_NiV_ 006	CN1C(=O)C23CC4=CC=CC(C4N2C(=O)C1(SS3)CO)O		6.83	6.56

			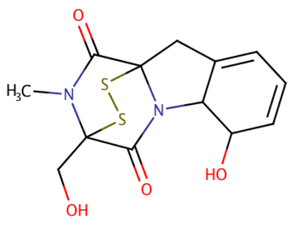		
7	Anti_NiV_007	CCN(CC)C1=CC=C(C=C1)C(=C2C=CC(=[N+](CC)CC)C=C2)C3=CC=CC=C3.OS(=O)(=O)[O-]	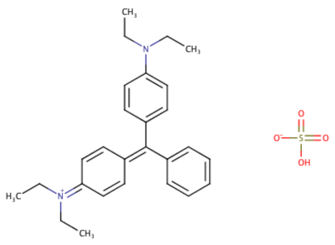	6.66	6.08
8	Anti_NiV_008	CCN1C(=S)S\C(=C/C2=CC=C(O2)C2=CC=CC=C2OC)C1=O	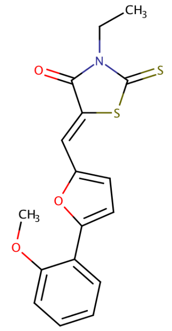	6.40	6.19
9	Anti_NiV_009	C1=CC=C2C(=C1)C=CC3=C2C=CC(=C3)C4=CC(=NN4C5=CC=C(C=C5)NC(=O)CN)C(F)(F)F	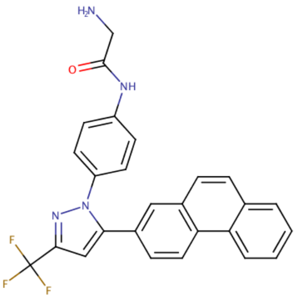	6.40	5.91
10	Anti_NiV_010	C(C1C(C(C(O1)C2=C(C(=NN2)C(=O)N)O)O)O)O	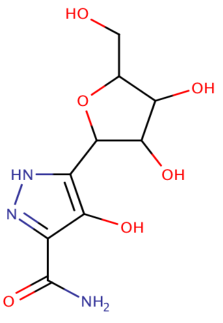	6.36	6.52

11	Anti_NiV_ 011	CCN1C(=O)S\C(=C/ C2=CC=C(O2)C2=C C=CC=C2[N+]([O-])=O)C1=S	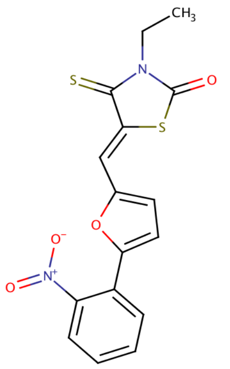	6.31	4.73
12	Anti_NiV_ 012	CN(C)C1=CC=C(C= C1)C(=C2C=CC(=[N +](C)C)C=C2)C3=CC =C(C=C3)N(C)C.[Cl-]	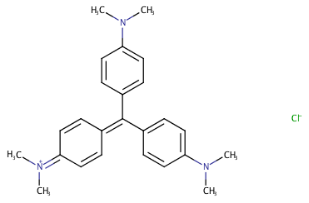	6.28	5.80
13	Anti_NiV_ 013	CCN(CC)CCCC(C)N C1=C2C=CC(=CC2= NC=C1)Cl	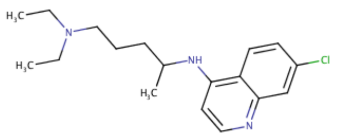	6.21	5.95
14	Anti_NiV_ 014	CCN1C(=O)/C(=C/c2 ccc(o2)c3ccccc3C(F) (F)F)/SC1=S	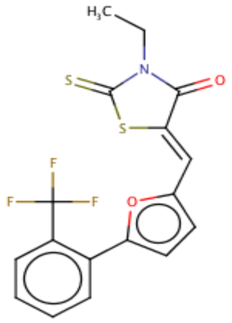	6.09	6.05
15	Anti_NiV_ 015	CCN1C(=O)C(=Cc2c cc(o2)c3ccccc3F)SC 1=S	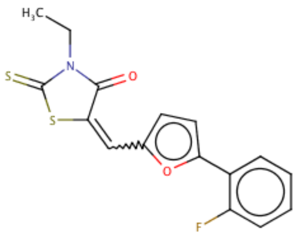	6.04	5.73
16	Anti_NiV_ 016	CCCN1C(=O)/C(=C/c 2ccc(o2)c3ccccc3)/S C1=S	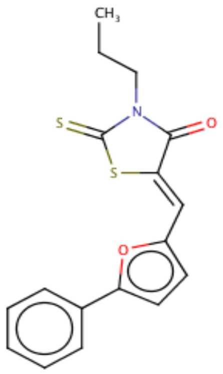	6.02	5.49

17	Anti_NiV_ 017	CCN1C(=O)/C(=C/c2 ccc(o2)c3ccccc3[N+](=O)[O-])/SC1=S	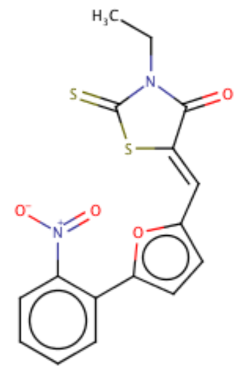	5.99	5.56
18	Anti_NiV_ 018	O=C1N(CC#C)C(=S) S\C1=C/C1=CC=C(O 1)C1=CC=CC=C1	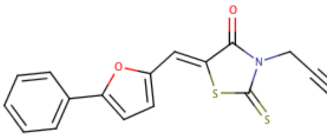	5.99	5.58
19	Anti_NiV_ 019	c1ccc(cc1)c2ccc(o2)/ C=C\3/C(=O)NC(=S) S3	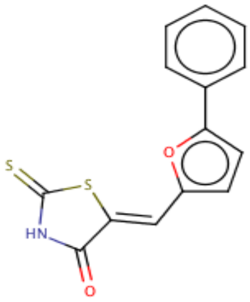	5.98	5.92
20	Anti_NiV_ 020	CCN1C(=S)S\C(=C/ C2=CC=C(O2)C2=C C=CC(OC)=C2)C1= O	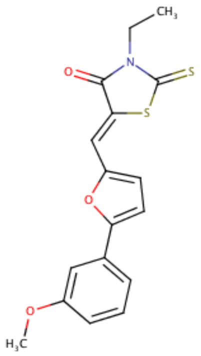	5.94	5.48
21	Anti_NiV_ 021	CN1C(=O)/C(=C/c2c cc(o2)c3ccccc3)/SC1 =S	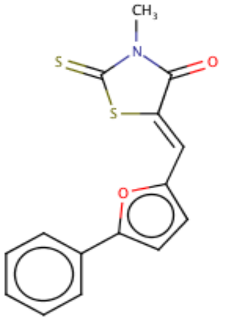	5.91	5.59

22	Anti_NiV_022	CCN1C(=O)/C(=C/c2ccc(o2)c3ccccc3Cl)/SC1=S	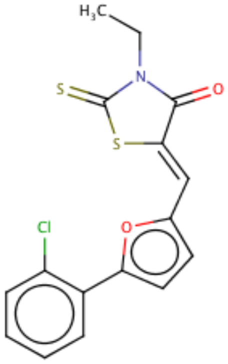	5.90	5.90
23	Anti_NiV_023	CCN1C(=O)/C(=C/c2ccc(o2)c3cccc(c3)Cl)/SC1=S	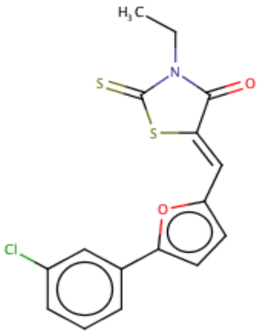	5.85	5.56
24	Anti_NiV_024	CCN1C(=O)/C(=C/c2ccc(o2)c3cccc(c3)F)/SC1=S	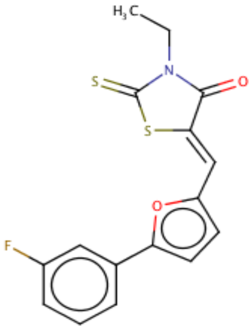	5.84	5.90
25	Anti_NiV_025	NC(=O)C1CCN(CC1)C1=CC2=C(C=C1F)C(=O)C(CN2C1CC1)C(=O)NCC1=CC=C(Cl)C=C1Cl	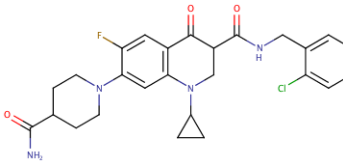	5.82	5.35
26	Anti_NiV_026	C1=CN(C(=O)N=C1N)C2C(C(C(O2)(CO)N=[N+]=[N-])O)O	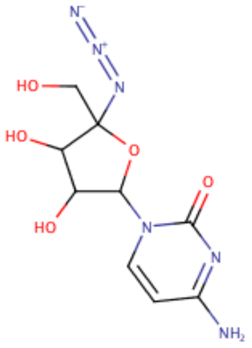	5.82	5.45
27	Anti_NiV_027	F[B-](F)(F)F.CCN1C(=S)S\C(=C/C2=CC=C(O2)C2=CC=CC=C2[N		5.79	5.89

		+]#N)C1=O	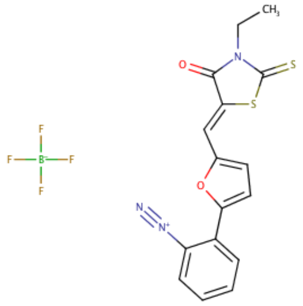		
28	Anti_NiV_ 028	OCCCN1C(=S)S\C(=C/C2=CC=C(O2)C2=CC=CC=C2)C1=O	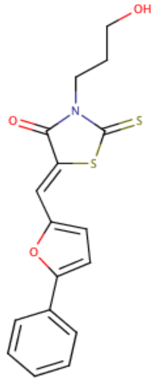	5.76	5.78
29	Anti_NiV_ 029	O=C(CCCC1SCC2NC(=O)NC12)OCCCN1C(=S)S\C(=C/C2=CC=C(O2)C2=CC=CC=C2)C1=O	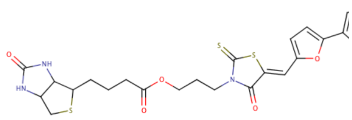	5.75	5.40
30	Anti_NiV_ 030	CCN1C(=O)/C(=C/c2ccc(o2)c3ccccc3)/SC1=S	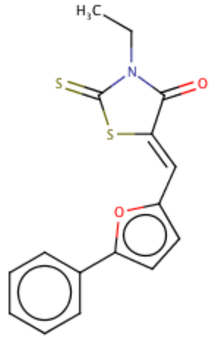	5.75	5.76
31	Anti_NiV_ 031	CC(=O)OCCCN1C(=S)S\C(=C/C2=CC=C(O2)C2=CC=CC=C2)C1=O	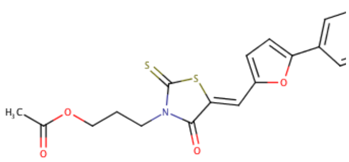	5.72	5.84

32	Anti_NiV_032	CCN1C(=S)S\C(=C/C2=CC=C(O2)C2=CC=CC=C2N)C1=O	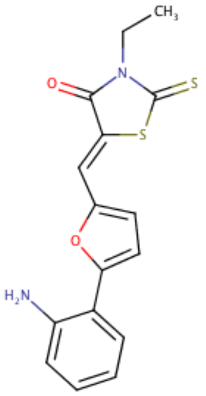	5.71	5.93
33	Anti_NiV_033	CCN1C(=S)S\C(=C/C2=CC=C(O2)C2=CC=CC=C2O)C1=O	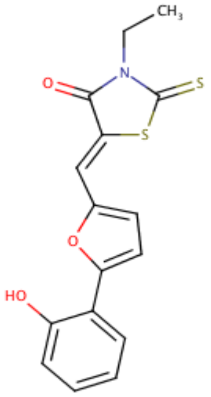	5.63	5.56
34	Anti_NiV_034	CCN1C(=S)S\C(=C/C2=CN=C(O2)C2=CC=CC=C2)C1=O	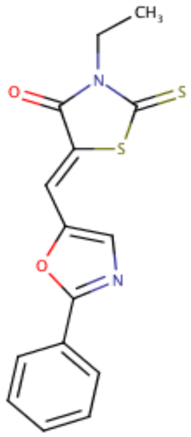	5.62	5.37
35	Anti_NiV_035	c1ccc(cc1)CN2C(=O)/C(=C/c3ccc(o3)c4ccccc4)/SC2=S	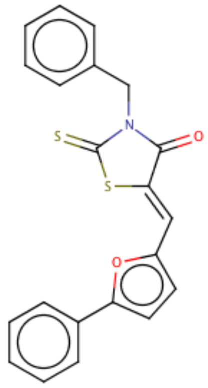	5.60	5.66
36	Anti_NiV_036	C=CCN1C(=O)C(=CC2=CC=C(O2)C3=CC=CC=C3)SC1=S		5.59	5.52

			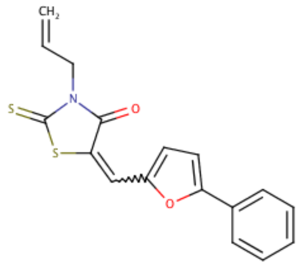		
37	Anti_NiV_037	CCN1C(=S)S\C(=C/C2=CC=C(S2)C2=CC=CC=C2)C1=O	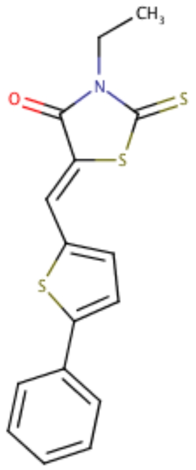	5.58	5.52
38	Anti_NiV_038	OCCN1CC(C(=O)NCC2=CC=C(Cl)C=C2Cl)C(=O)C2=C1C=C(N1CCC(CC1)C(=O)C1CO1)C(F)=C2	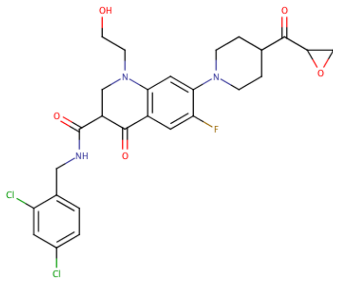	5.52	5.46
39	Anti_NiV_039	FC1=CC2=C(C=C1N1CCN(CC1)C(=O)C1CO1)N(CC(C(=O)NCC1=CC=C(Cl)C=C1Cl)C2=O)C1CC1	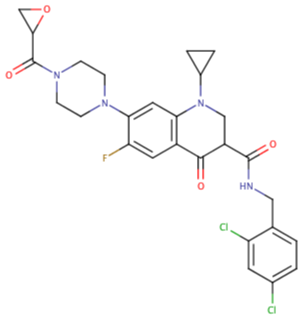	5.52	5.41
40	Anti_NiV_040	C1=CC=C(C=C1)C(C2=CC=CC=C2)(C3=CC=CC=C3Cl)N4C=CN=C4	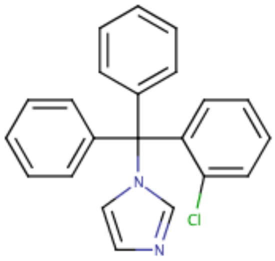	5.46	4.68
41	Anti_NiV_041	CC(C)NCC(COC1=CC=CC2=CC=CC=C2		5.42	5.34

		1)O	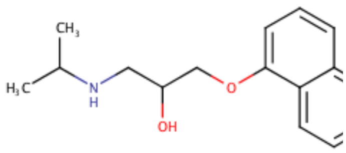		
42	Anti_NiV_042	c1ccc2c(c1)CCC(=C2)S(=O)(=O)Nc3ccc(cc3)n4cnnn4	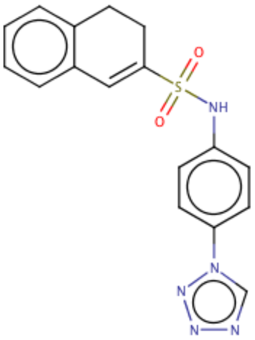	5.41	5.89
43	Anti_NiV_043	OCCN1CC(C(=O)NCC2=CC=C(Cl)C=C2Cl)C(=O)C2=C1C=C(N1CCN(CC1)C(=O)C1CO1)C(F)=C2	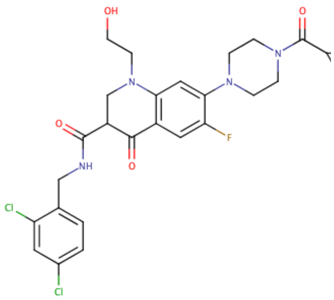	5.40	5.42
44	Anti_NiV_044	C1=NC(=NN1C2C(C(C(O2)CO)O)O)C(=O)N	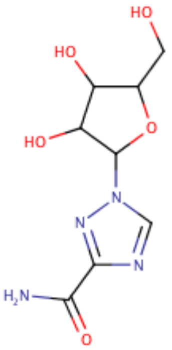	5.38	5.94
45	Anti_NiV_045	CC(CCC(=O)O)CC(C)CC(C)C(=O)C=C(C(C)CC(C)CC=CC(C)C(C(C)C(CC1CCC(O1)(C)C2CCC(O2)(C)C(C)O)O)O)O	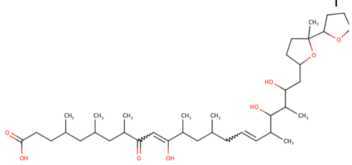	5.28	4.52

46	Anti_NiV_046	CCN(C1=NC(=C(N=C1Cl)C(=O)N=C(N)N)N)C(C)C	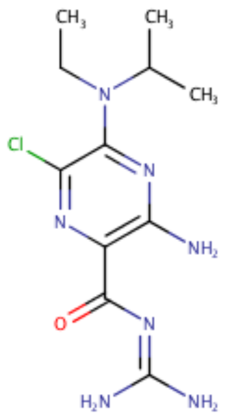	5.22	4.45
47	Anti_NiV_047	CC(C)C1C2=C(CCC1(CCN(C)CCCC3=NC4=CC=CC=C4N3)OC(=O)COC)C=C(C=C2)F	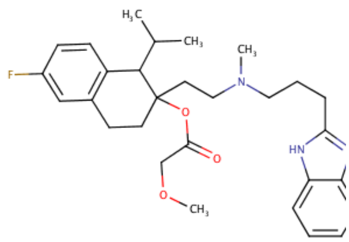	5.21	5.64
48	Anti_NiV_048	CC(COC1=CC=CC=C1)N(CCCl)CC2=CC=CC=C2	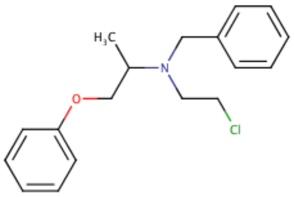	5.16	4.51
49	Anti_NiV_049	C1CCCC2CCN3CCCC(C3O2)CCCCCCC4CCN5CCCC(C5O4)CC1	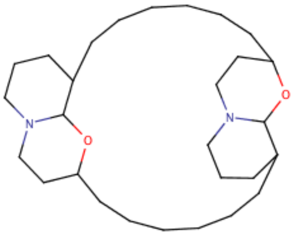	5.15	4.26
50	Anti_NiV_050	CCC(=C(C1=CC=C(C=C1)O)C2=CC=C(C=C2)OCCN(C)C)C3=CC=CC=C3	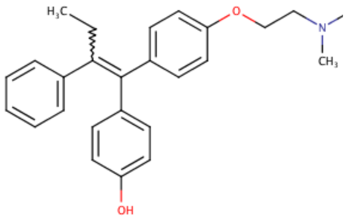	5.12	4.81
51	Anti_NiV_051	CN(C)S(=O)(=O)c1ccc2c(c1)CCN2S(=O)(=O)c3cccs3		5.11	5.06

			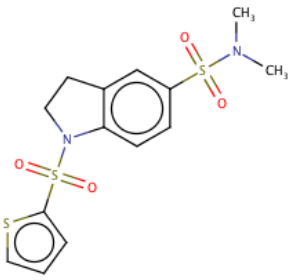		
52	Anti_NiV_ 052	OCCN1C=C(C(=O)N CC2=CC=C(Cl)C=C2)C(=O)C2=C1C=C(C =C2)C(F)(F)F	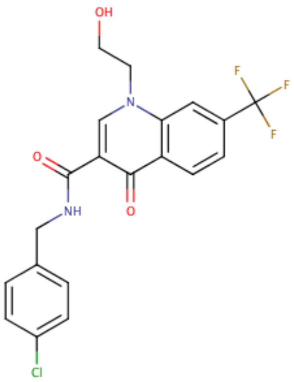	5.10	5.58
53	Anti_NiV_ 053	OC(=O)C1=CC2=C(NC=C(C(O)=O)C2=O)C=C1	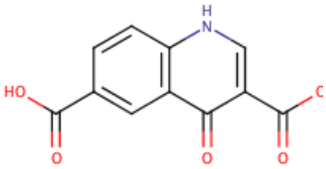	5.10	4.89
54	Anti_NiV_ 054	C1=CC=C(C=C1)CC 2=NC3=C(O2)C=CC(=C3)N	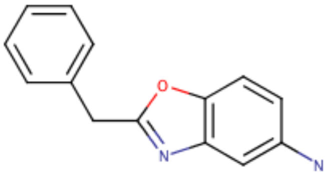	5.10	5.27
55	Anti_NiV_ 055	Fc1ccc(cc1F)S(=O)(= O)Nc2ccc(cc2)n3nnn c3	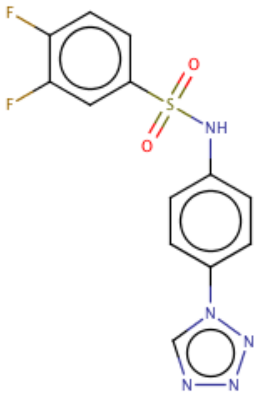	4.93	5.09

56	Anti_NiV_056	CC(C)(C)C1=CC(=C(C=C1O)C(C)(C)C)O	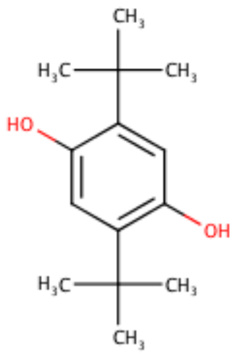	4.91	4.75
57	Anti_NiV_057	CCC1(CCC(O1)C2(CCC3(O2)CC(C(C(O3)C(C)C(C(C)C(=O)O)OC)C)O)C)C4C(CC(O4)C5C(CC(C(O5)(CO)O)C)C)C	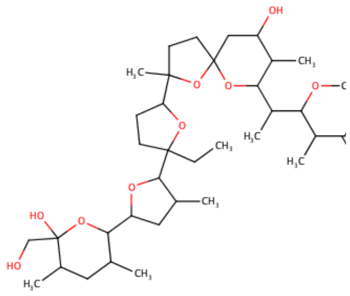	4.91	4.67
58	Anti_NiV_058	C1C(=O)NC(=O)N1N=CC2=CC=C(O2)C3=CC=C(C=C3)[N+](=O)[O-]	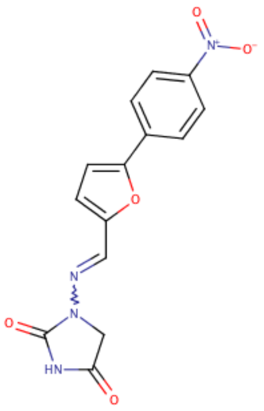	4.91	4.38
59	Anti_NiV_059	CCC1C(=O)N(CC(=O)N(C(C(=O)NC(C(=O)N(C(C(=O)NC(C(=O)NC(C(=O)N(C(C(=O)N(C(C(=O)N(C(C(=O)N(C(C(=O)N1)C(C(C)CC=CC)O)C)C(C)C)C)CC(C)C)C)CC(C)C)C)C)C)CC(C)C)C)C(C)C)CC(C)C)C)C	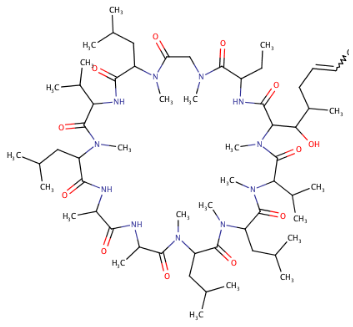	4.90	4.38

60	Anti_NiV_060	C1=C(N=C(C(=O)N1)C(=O)N)F	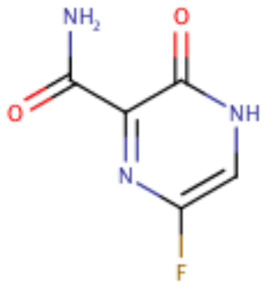	4.83	4.34
61	Anti_NiV_061	CC1=C(C(=C(C(=C1O)C(=O)C)O)CC2=C(C3=C(C(=C2O)C(=O)C=CC4=CC=CC=C4)OC(C=C3)(C)C)O)O	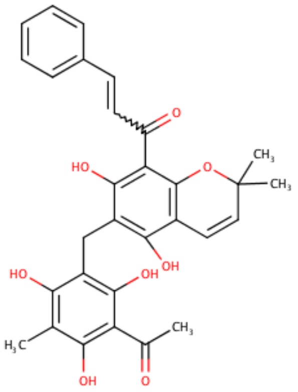	4.70	4.74
62	Anti_NiV_062	CCOC(=O)C1C(N(CC=C)C(C2=CC=C(C=C2)[N+]([O-])=O)C(C(=O)OCC)=C1O)C1=CC=C(C=C1)[N+]([O-])=O	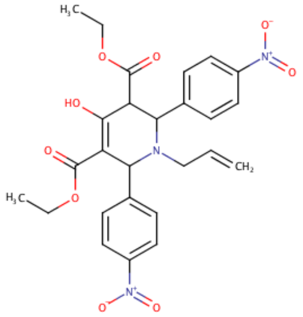	4.70	5.17
63	Anti_NiV_063	CC(C)C(CCCN(C)CCC1=CC(=C(C=C1)OC)OC)(C#N)C2=CC(=C(C=C2)OC)OC	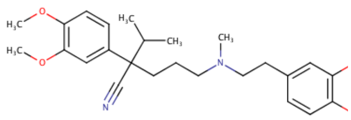	4.66	4.71
64	Anti_NiV_064	CC(CCCC(C)(C)O)C1CCC2C1(CCCC2=CC=C3CC(CCC3=C)O)C	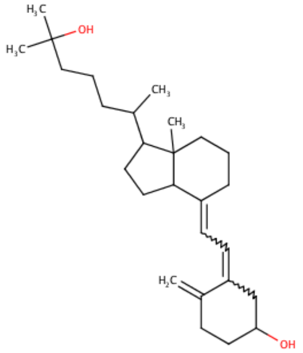	4.51	4.59
65	Anti_NiV_065	C1CCC(CC1)C(=O)N2CC3C4=CC=CC=C4CCN3C(=O)C2		4.47	4.88

			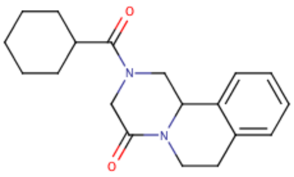		
66	Anti_NiV_066	C1CC(N(C1)C(=O)C2CCCN2C(=O)C(CCCN=C(N)N)N)C(=O)NCC(=O)NC(CC3=CC=CC=C3)C(=O)NC(CO)C(=O)N4CCCC4C(=O)NC(CC5=CC=CC=C5)C(=O)NC(CCCN=C(N)N)C(=O)O	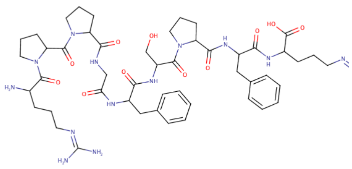	4.47	4.73
67	Anti_NiV_067	CC12CCC3C(C1CCC2NCCCCCCCN4C(=O)C=CC4=O)CCC5=C3C=CC(=C5)OC	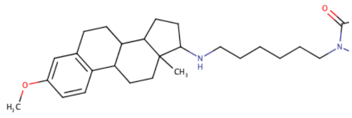	4.44	4.96
68	Anti_NiV_068	C1=CC=C(C=C1)C2(C(=O)NC(=O)N2)C3=CC=CC=C3	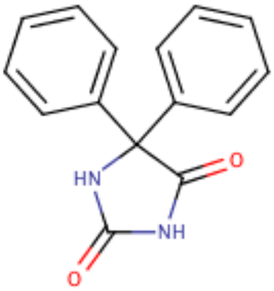	4.29	4.30
69	Anti_NiV_069	CC(C)CCCC(C)C1CCC2C1(CCCC2=CC=C3CC(CCC3=C)O)C	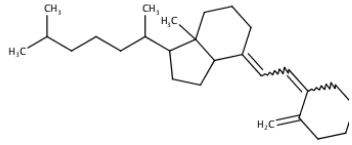	4.25	4.57
70	Anti_NiV_070	C1=CC=C(C=C1)CN=C(N)NC(=O)C2=C(N=C(C(=N2)Cl)N)N	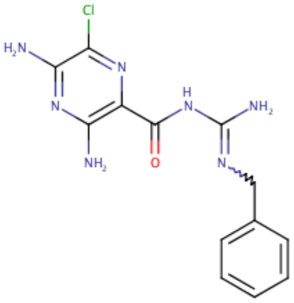	4.19	4.75
71	Anti_NiV_071	CC1CCC2(C3(CC4(C5(C(C(C3(C5(C2(C1O)O4)O)O)OC(=O)C6=CC=CN6)(C(C)C)O)C)O)C)O		4.18	5.21

			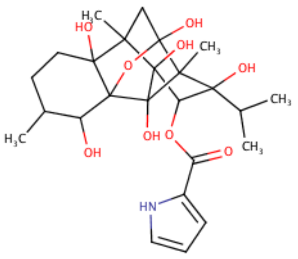		
72	Anti_NiV_ 072	CCN1C(=O)/C(=C/c2 ccc(o2)C)/SC1=S	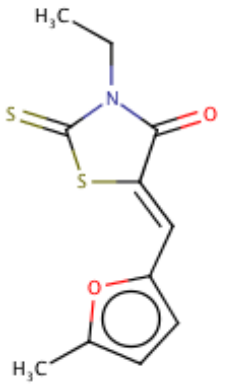	4.00	4.16
73	Anti_NiV_ 073	CCN1C(=O)/C(=C/c2 ccco2)/SC1=S	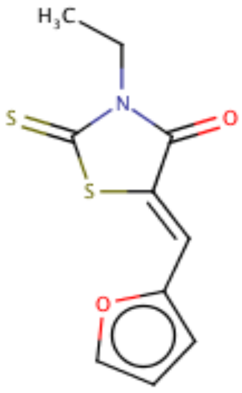	4.00	4.74
74	Anti_NiV_ 074	CCN1C(=S)S\C(=C/ C2=CC=CC(=C2)C2 =CC=CC=C2)C1=O	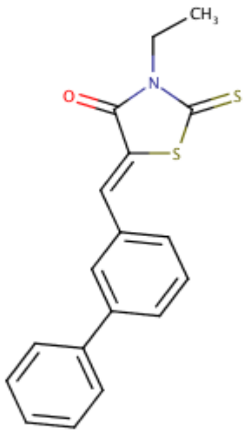	4.00	5.67

75	Anti_NiV_ 075	C=CN1C(=O)/C(=C/c 2ccc(o2)c3ccccc3)/S C1=S	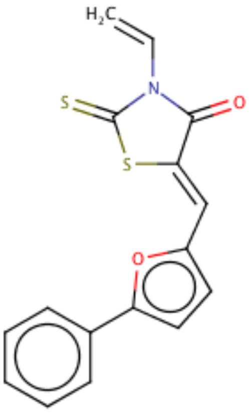	4.00	5.16
76	Anti_NiV_ 076	CCN1C(=O)/C(=C/c2 ccc(o2)c3ccccc3[N+](=O)[O-])/SC1=O	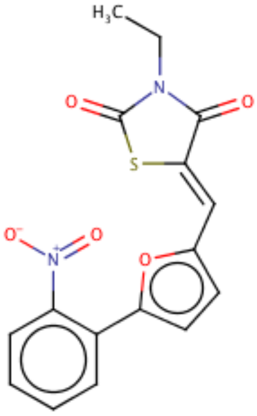	4.00	4.84
77	Anti_NiV_ 077	C=CN1C(=S)S\C(=C/ C2=CC=C(O2)C2=C C=CC=C2)C1=S	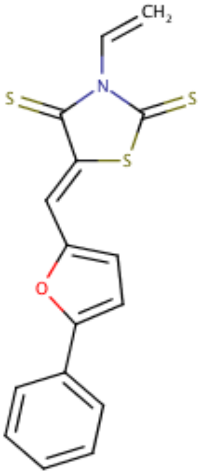	4.00	5.38
78	Anti_NiV_ 078	CCN1C(=S)S\C(=C/ C2=CC=C(O2)C2=C C=CC=C2[N+]([O-])=O)C1=S	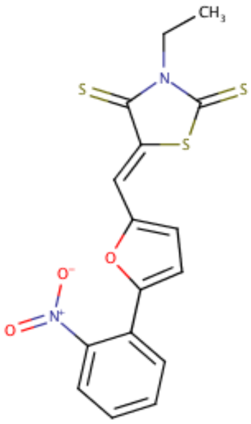	4.00	5.25

79	Anti_NiV_079	C=CCN1C(=S)SC(CC2=CC=C(O2)C2=CC=CC=C2)C1=O	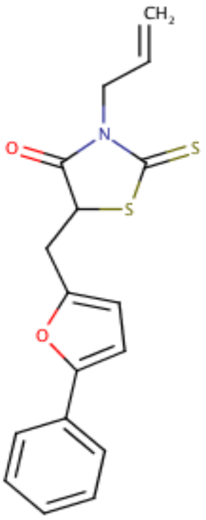	4.00	5.32
80	Anti_NiV_080	CC(=O)NCCCN1C(=O)S\C(=C/C2=CC=C(O2)C2=CC=CC=C2)C1=O	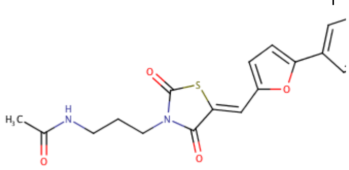	4.00	4.63
81	Anti_NiV_081	NCCCN1C(=O)S\C(=C/C2=CC=C(O2)C2=CC=CC=C2)C1=O	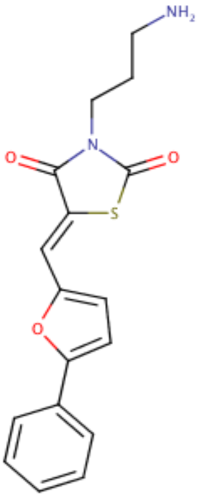	4.00	5.42
82	Anti_NiV_082	CC(C)COC(=O)NCCCN1C(=S)S\C(=C/C2=CC=C(O2)C2=CC=CC=C2)C1=O	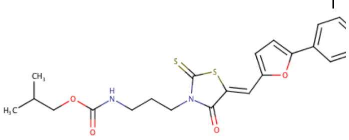	4.00	5.38
83	Anti_NiV_083	O=C(CCCC1SCC2NC(=O)NC12)OCCCN1C(=O)S\C(=C/C2=CC=C(O2)C2=CC=CC=C2)C1=O	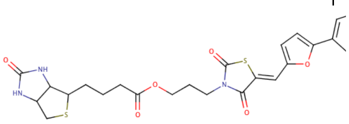	4.00	4.19
84	Anti_NiV_084	B(C1=CC=CC=C1)(C2=CC=CC=C2)OCCN		3.88	3.84

			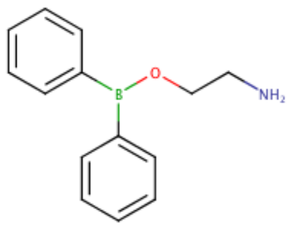		
85	Anti_NiV_085	CC1=C(C(C(=C(N1)C)C(=O)OC)C2=CC=CC=C2[N+](=O)[O-])C(=O)OC	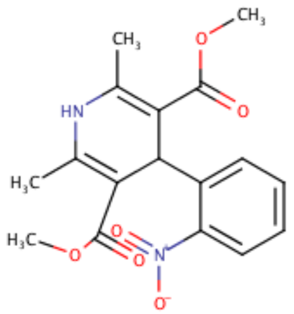	3.61	3.74
86	Anti_NiV_086	CCCCCCCCC1=C2C3=C(C=C1)N(C(C(=O)NC(CC3=CN2)CO)C(C)C)C	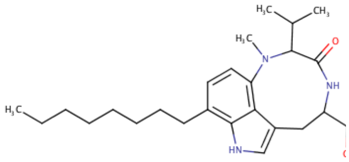	3.51	4.51
87	Anti_NiV_087	CCOC(=O)CCCCCCCCCCCN(C)C(=O)COC(C)C(C)OCC(=O)N(C)CCCCCCCCCCCC(=O)OCC	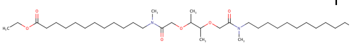	3.46	4.20
88	Anti_NiV_088	CCCCCCCCCCCCCC(=O)OC1C(C2(C(C=C(CC3(C2C=C(C3=O)C)O)CO)C4C1(C4(C)C)OC(=O)C)O)C	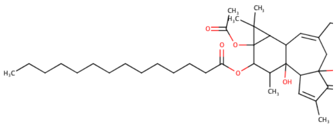	3.43	3.28
89	Anti_NiV_089	C1CCC(CC1)(CC(=O)O)CN	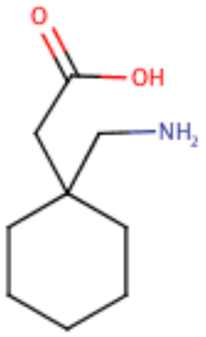	3.27	4.28
90	Anti_NiV_090	CCOC(=O)C1=C(NC(=C(C1C2=CC=CC=C2Cl)C(=O)OC)C)COCCN.C1=CC=C(C=C1)S(=O)(=O)O	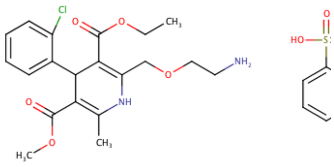	3.19	3.50
91	Anti_NiV_091	CN1CCC2=CC(=C3C=C2C1CC4=CC=C(		3.18	4.00

		C=C4)OC5=C(C=CC(=C5)CC6C7=C(O3)C(=C(C=C7CCN6C)OC)OC)OC)OC	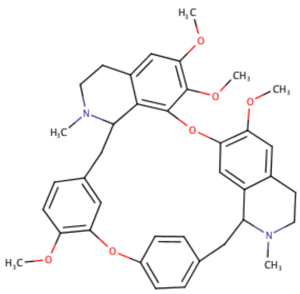		
92	Anti_NiV_092	C1(=C(N=C(C(=N1)Cl)N)N)C(=O)N=C(N)N	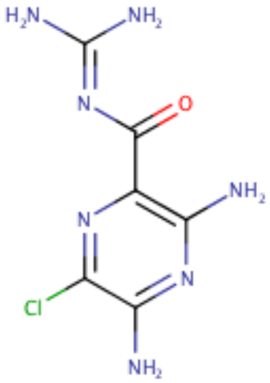	3.18	4.19
93	Anti_NiV_093	Cl[La](Cl)Cl	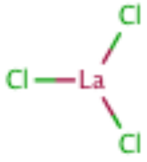	2.99	2.48
94	Anti_NiV_094	CCCCCCCCC=CCCCCCCCC(=O)O	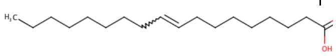	2.73	3.08
95	Anti_NiV_095	CN1C=NC2=C1C(=O)N(C(=O)N2C)C	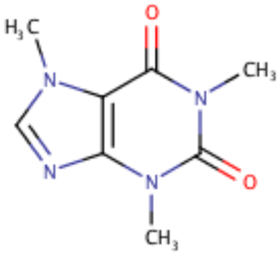	2.12	3.22

Supplementary Table S3. Details of prediction done using 74 decoy set through prediction model employing support vector machine along with their structures

**S.No.**	**ZINC_ID**	**SMILES**	**Images**	**Predicted_pIC50**
**1**	ZINC01418565	Nc1nc(N)n2c(c1)ncn2	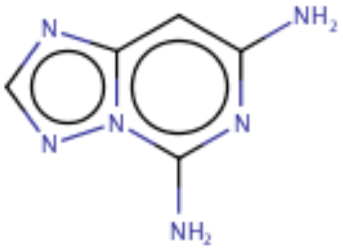	3.8867641
2	ZINC76045055	Cc1n[nH]c(n1)[C@H]1OCCN(C1)C(=O)c1cc(sc1Cl)Cl	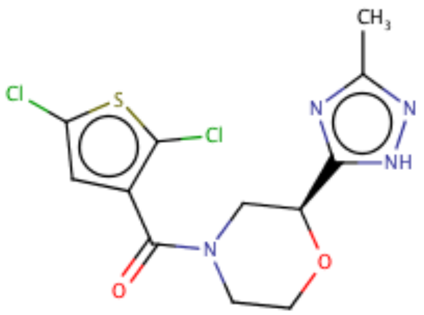	5.8360873
3	ZINC94666930	Brc1ccc(s1)S(=O)(=O)[N-]c1ccc([nH+]c1C)C	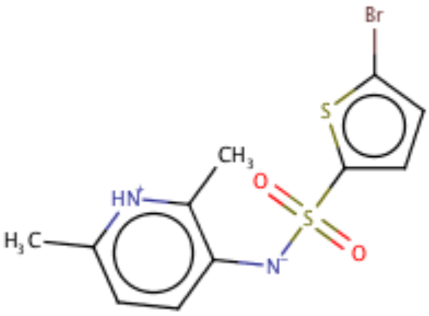	5.4610064
4	ZINC61718549	FC(c1nnc2n1nc(s2)c1noc(c1)c1ccco1)(Cl)F	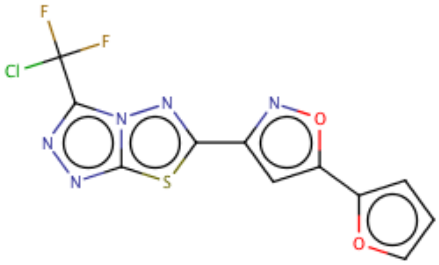	5.8472315

5	ZINC49551322	O=C(c1cc2CCCc2[nH]c1=O)N1CCCCC1	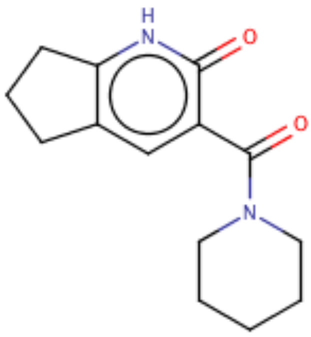	4.729709
6	ZINC32909472	O=C(NCC1CC1)NCCc1cccc(c1)Cl	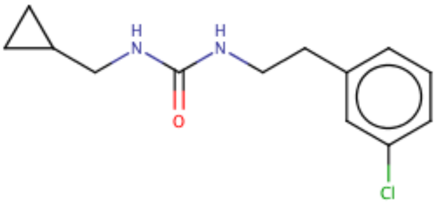	4.3834935
7	ZINC92779969	O=C([C@H]1NCc2c(C1)nc[nH]2)Nc1c(C)n[nH]c1C	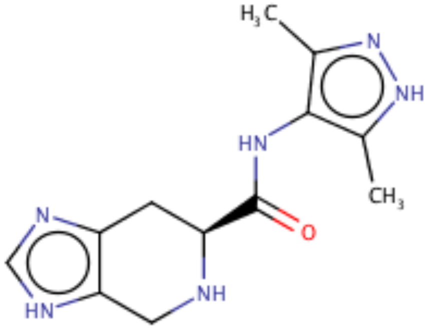	5.7719926
8	ZINC08655749	COC(=O)c1nc2nnc(n2c(c1)c1ccc(cc1)N)N	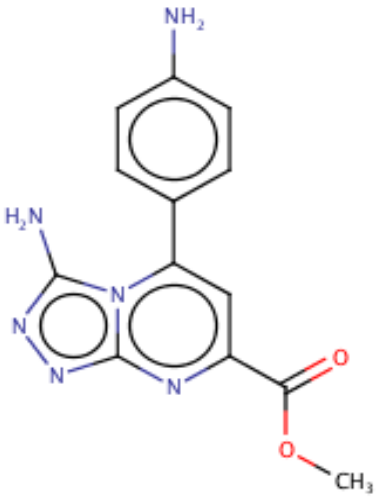	6.019685

9	ZINC40162136	CCN(C(=O)c1ccc2c(c1)[nH]c(=O)c(=O)[nH]2)Cc1ccc(s1)Cl	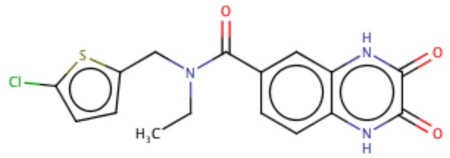	5.3902657
10	ZINC19846561	N#Cc1cccnc1Sc1nc2c(s1)cccc2	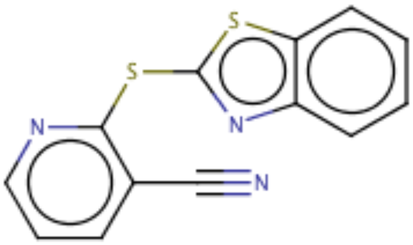	5.0631296
11	ZINC28802886	N#Cc1c(NC(=O)CSc2scc(n2)C)sc2c1CCCCC2	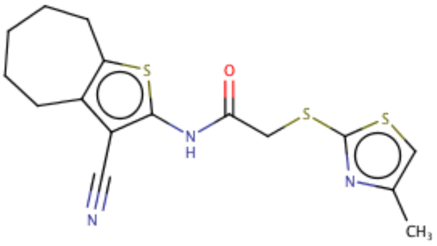	6.863947
12	ZINC07780252	Cc1csc(n1)Sc1nc(nc2c1cccc2)c1cccs1	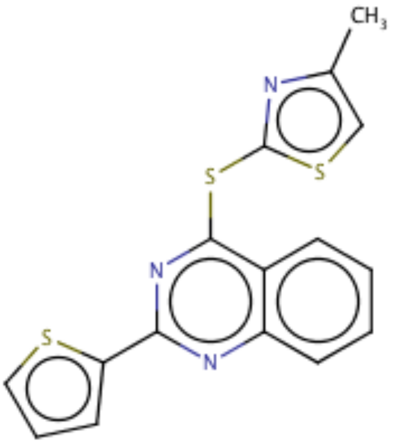	4.6468422

13	ZINC16940346	OC[C@@H]1O[C@@H]([C@@H]([C@H]1O)O)[C@@H]1C=Nc2c1ncnc2N	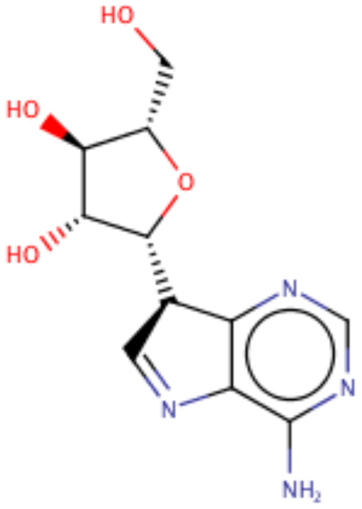	6.0687112
14	ZINC70807776	N#CC1(CCCC1)C(=O)Oc1ccc(c(c1)C)C	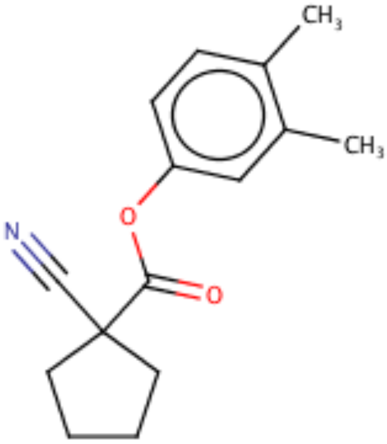	5.1389496
15	ZINC93584610	CN(Cc1cccn1C)Cc1cccc2c1N(CC2)C(=O)OC(C)(C)C	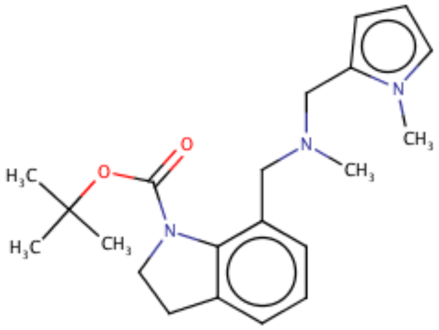	4.2884885

16	ZINC94975524	Nc1cc(F)c(c(c1)F)SCc1scc(n1)C(C)(C)C	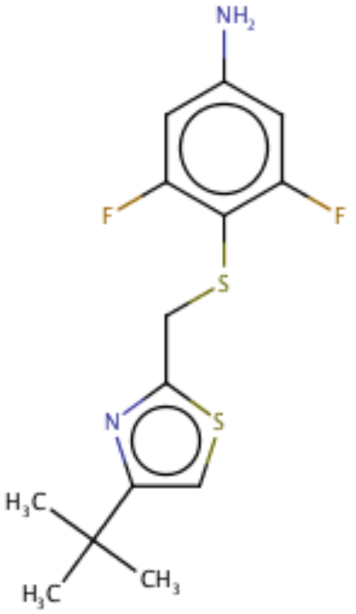	4.3705865
17	ZINC94541485	Nc1n[nH]cc1CNS(=O)(=O)c1cc(c(s1)Br)C	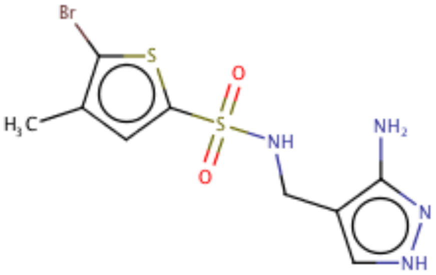	6.0091462
18	ZINC92711562	c1ccc2c(c1)S[C@H](C2)CSc1nc2c(s1)cccc2	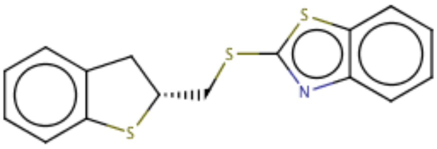	4.9740363
19	ZINC02666989	CC(CC(=O)N[C@@H](C(Cl)(Cl)Cl)Nc1cccc(c1Cl)Cl)C	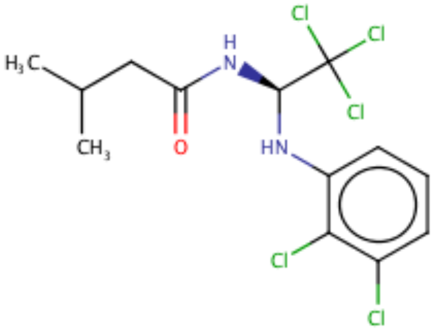	4.9088033

20	ZINC81462747	CCN(C(=O)c1ccc(c(c1)S(=O)(=O)N)C)c1scc(n1)C	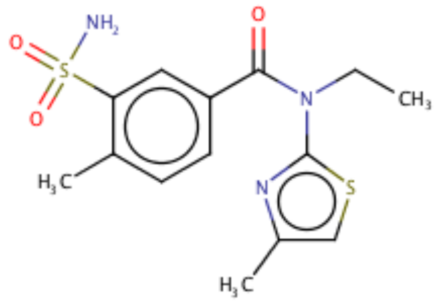	4.7152084
21	ZINC28304734	Cc1[nH]/c(=N\S(=O)(=O)c2ccc(cc2)C(C)(C)C)/sc1C	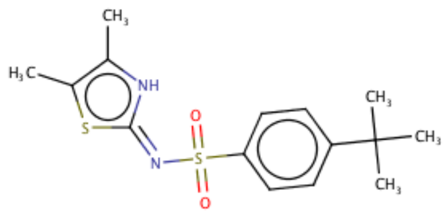	4.7252084
22	ZINC85235369	CCc1ccc(s1)S(=O)(=O)[N-]c1c[nH+]ccc1C	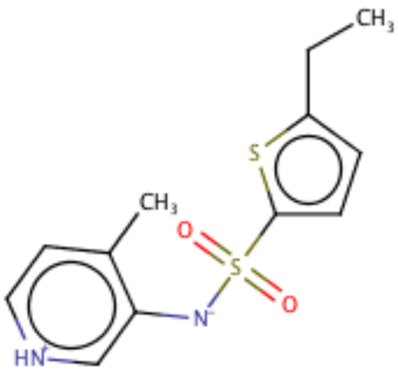	5.8046097
23	ZINC40212035	CC(N(C(=O)Cn1c(CCCCCNC(=O)C(C)(C)C)nc2c1cccc2)Cc1ccccc1)C	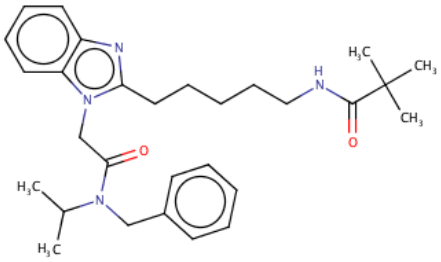	4.6624828
24	ZINC84509637	COCc1c(sc2c1c(F)ccc2)c1onc(n1)c1cnccn1	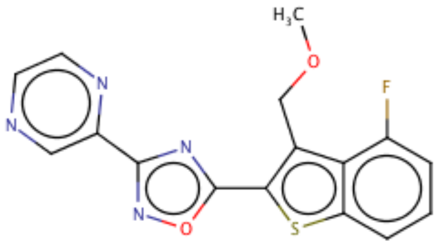	5.4898942

25	ZINC09145092	CCc1cccc(c1NC(=O)CSc1nnc(n1C1CCCCC1)c1cccc(c1)F)C	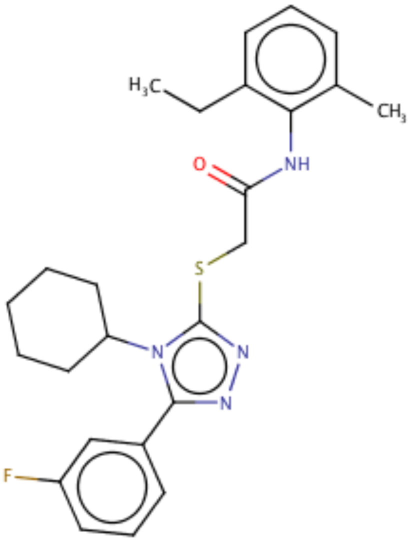	5.2362261
26	ZINC89447997	CCc1onc(c1NC(=O)c1cc(C)c(c(c1)S(=O)(=O)NC)C)C	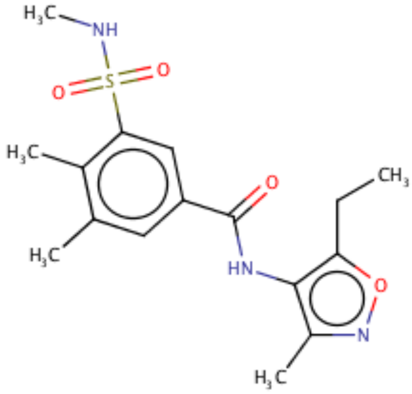	4.7440007
27	ZINC12428336	OC[C@H]([C@H](c1cnc2c(n1)c(=O)[nH]c(n2)N)O)O	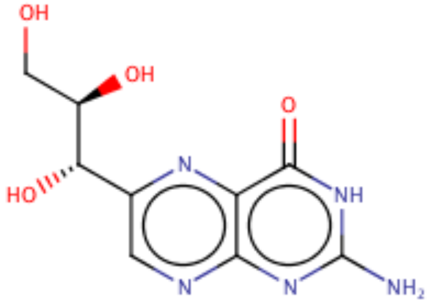	4.9369192

28	ZINC41107492	Fc1cccc(c1)NC(=O)c1cccnc1Br	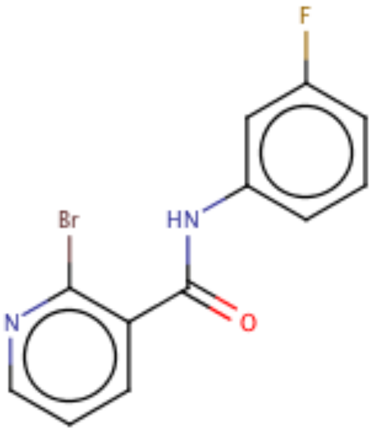	4.757407
29	ZINC00192078	COc1ccc2c(c1)ccc(c2)S(=O)(=O)Nc1ccccn1	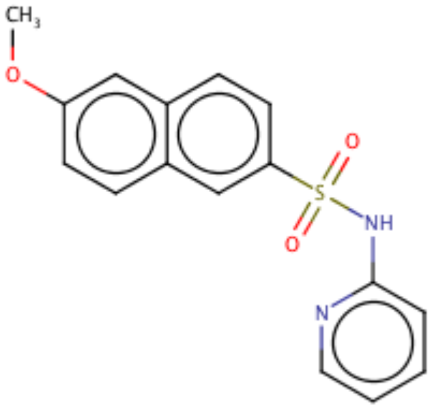	5.7803614
30	ZINC71883568	Cc1cn(nc1NC(=O)c1noc(c1)c1ccco1)c1ccccc1	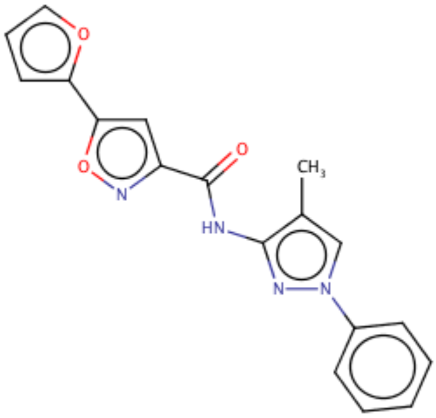	5.6745762
31	ZINC94883725	Fc1ccc2c(c1)sc(n2)NCc1sccc1C	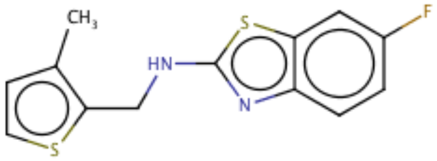	5.5519799

32	ZINC16952310	CSc1[nH]c(=O)c2c(n1)n(nn2)[C@H]1OC[C@@H]([C@H]([C@@H]1O)O)O	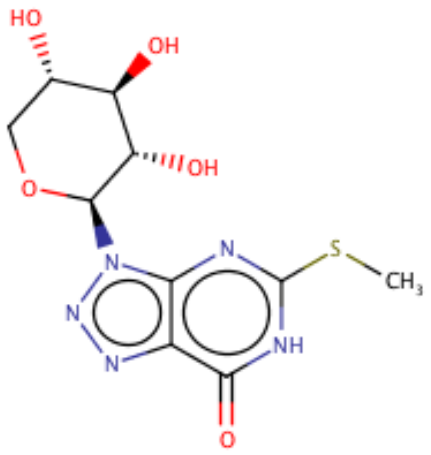	6.4557595
33	ZINC24257777	COC(=O)c1cccc(c1)S(=O)(=O)Nc1cc(C)ccc1F	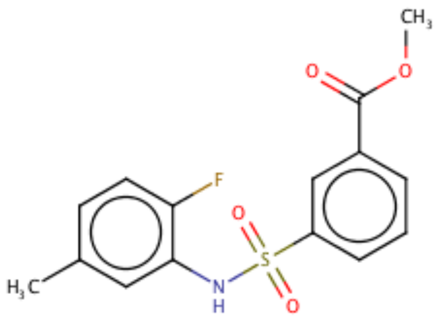	5.3899743
34	ZINC72481528	O=c1cc(nc([nH]1)N)N1CCN(CC1)c1ncnc2c1c(C)nn2C	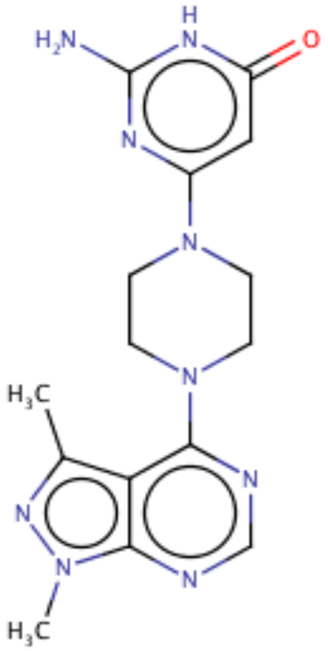	4.4301798

35	ZINC04260552	OC[C@]1(C)[C@H](O)CC[C@@]2([C@@H]1Cc1sc(nc1[C@H]2CC(=O)Nc1ccccc1O)c1cccnc1)C	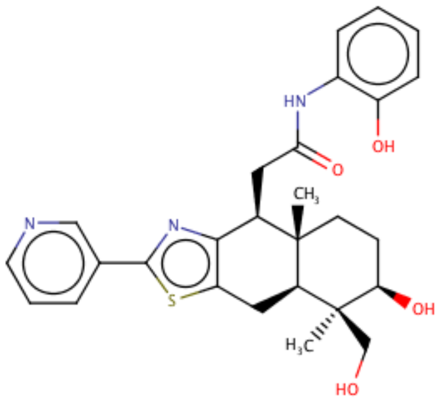	5.9589788
36	ZINC20496761	N#CC1(CCCC1)c1ccc(cc1)NC(=O)c1ccc(o1)c1cccc(c1Cl)Cl	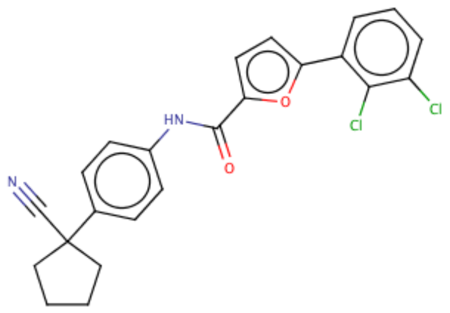	6.2292559
37	ZINC10555431	Fc1ccc(cc1)n1nc(nc1C)C(=O)Nc1scc(n1)C	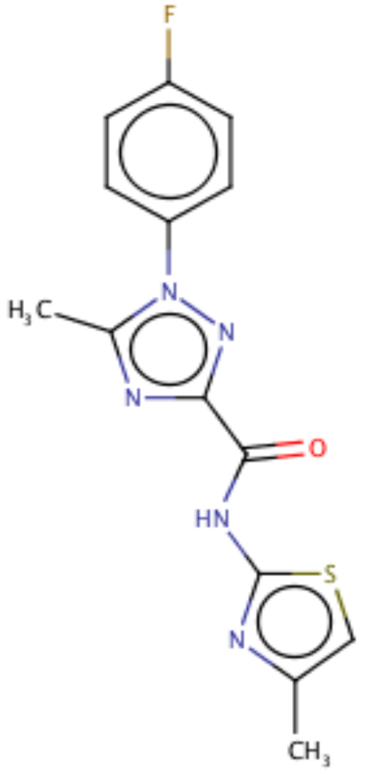	4.8477151

38	ZINC80529481	CN(C(=O)c1ccc(cc1)SC(F)(F)F)Cc1nccs1	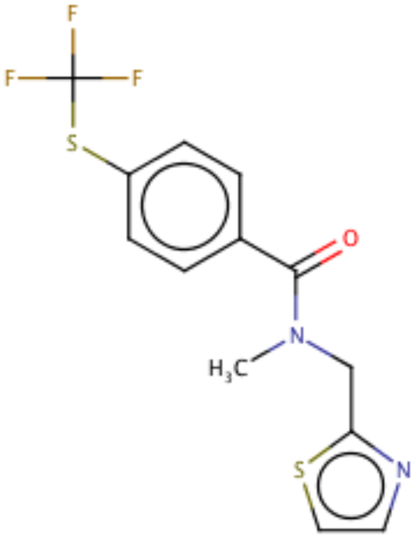	3.8880513
39	ZINC09670144	O=C(Nc1ccc(cc1)NS(=O)(=O)c1ccc(c(c1)F)F)CCc1nc2ccccc2c(=O)[nH]1	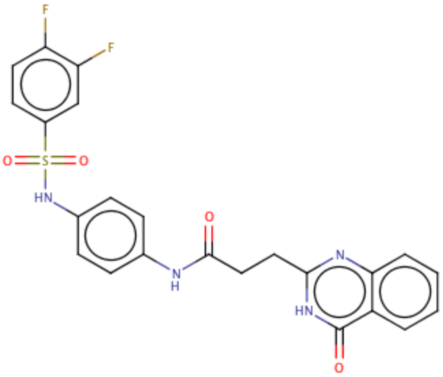	5.2087045
40	ZINC07006610	CCC(=O)Nc1ccc(cc1)C1=Nn2c(SC1)nnc2c1cccc(c1)F	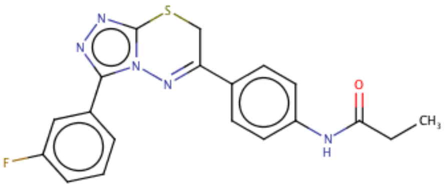	5.2481278
41	ZINC82108957	O=C(N1CCCC[C@@H]1c1nccs1)c1csc(n1)c1cccs1	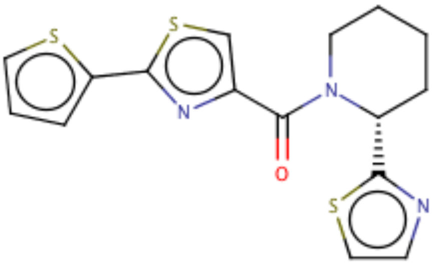	4.8600598
42	ZINC48561162	O=C(Nc1ccc(c(c1)Cl)N1CCNC(=O)C1)NCCc1ccc(cc1)F	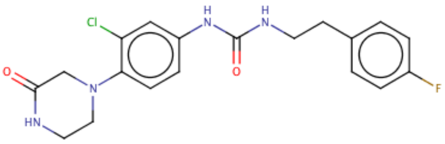	5.1645088

43	ZINC72646183	O=C([C@@H]1C[C@@H]2O[C@H]1CC2)Nc1scc(n1)c1ccc2c(c1)OCO2	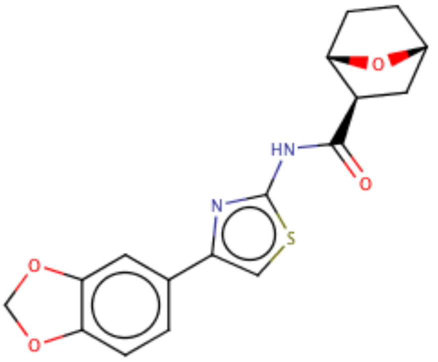		4.7391872
44	ZINC92664040	N#Cc1c(Cl)nsc1N([C@H](c1ccc(cc1)[S@@](=O)C)C)C	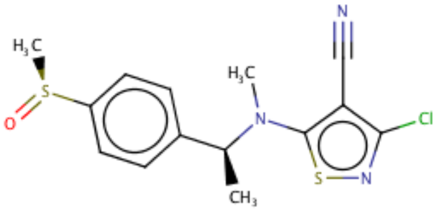		5.6224617
45	ZINC12955489	O=c1cc(c2c([nH]1)c(C)ccc2)C(F)(F)F	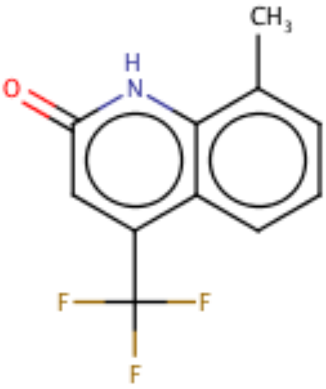		4.9421349
46	ZINC05115341	OC[C@H]1O[C@H]([C@@H]([C@@H]1O)O)n1cnc2c1nc([nH]c2=O)N(C)C	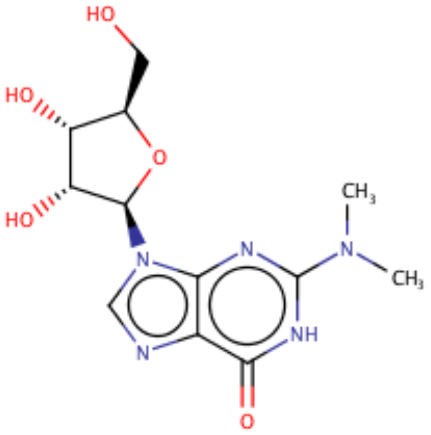		5.3034263

47	ZINC08593803	Cc1cc(C)c(c(c1)C)NC(=O)Cn1c2ccccc2c(=O)n(c1=O)CCC(=O)N1CCc2c(C1)cccc2	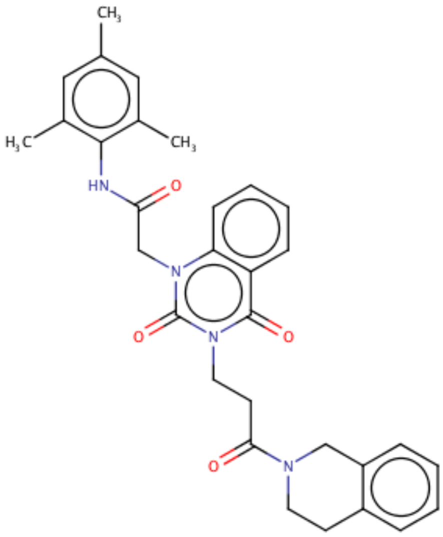	4.8599469
48	ZINC64953026	CCCN1C(=O)c2n(C[C@@]1(C)C(=O)NCc1ccc(cc1)C)cnc2C(=O)N1CCN(CC1)c1ccccn1	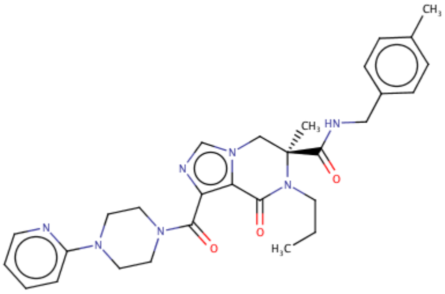	4.473165
49	ZINC19560635	Cc1nc(c(s1)c1cc(=O)[nH]c(n1)NCc1cccs1)C	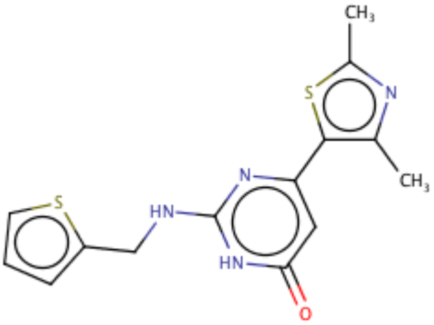	5.2568595
50	ZINC19596883	COc1ccc(cc1)n1nnnc1c1cnc2c(c1N)ccc(c2)Cl	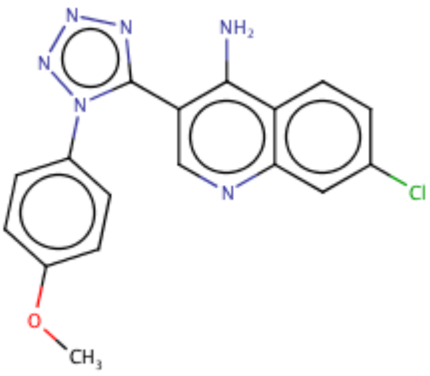	6.296559

51	ZINC12375827	Clc1nss/c/1=N\c1ccccc1	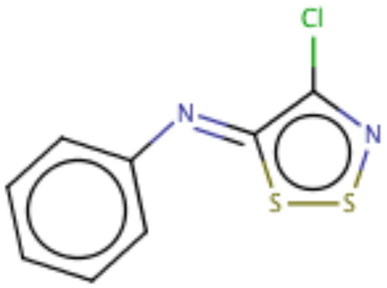	4.2259402
52	ZINC05998403	Clc1ccccc1CS(=O)(=O)c1ncn[nH]1	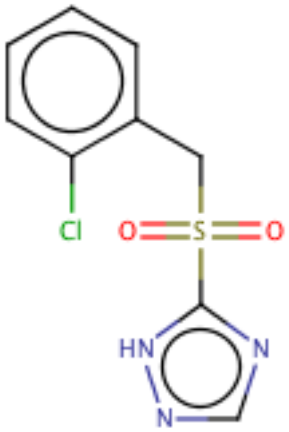	5.2383476
53	ZINC03378586	CCOC(=O)c1c(C)[nH]c(c1C)C(=O)CSc1nnc(s1)Nc1ccc(cc1)SC(F)F	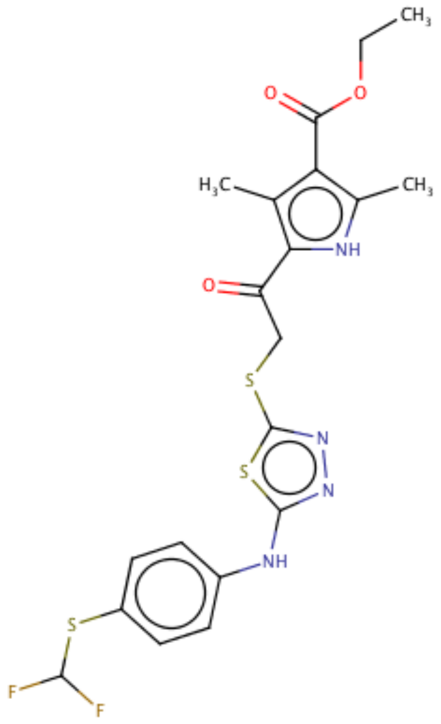	4.4241811

54	ZINC05309880	CCCC[C@]([C@H](c1ccccc1)C=C)(c1ccccc1)O	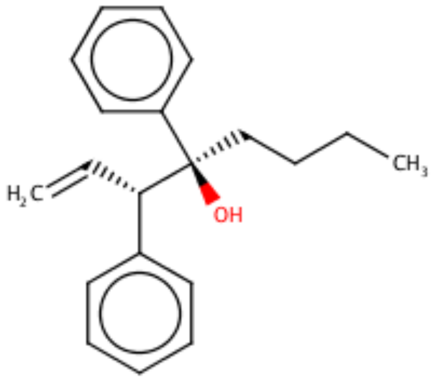	4.7219278
55	ZINC89297038	N#C[C@H](Cc1cccs1)COC(=O)c1ccc(cc1)[S@](=O)C(C)C	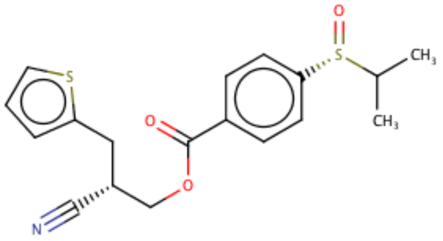	5.981777
56	ZINC23156662	O=C(C1c2ccccc2Oc2c1cccc2)Nc1nnc(o1)c1ccco1	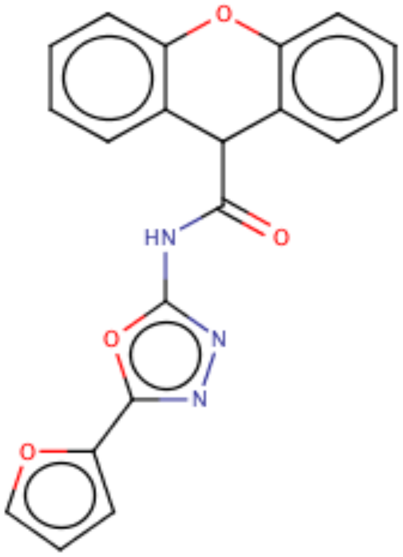	6.0546415
57	ZINC00440729	CC(=C)CSc1nc2sc3c(c2c(=O)n1CC(=C)C)CCC3	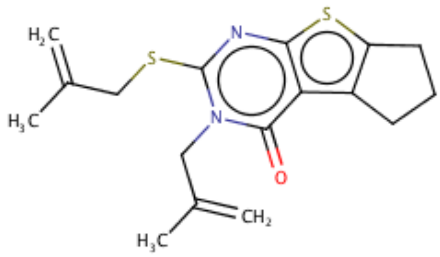	5.9091266

58	ZINC07059876	Clc1ccc2c(c1)c(=O)[nH]c(n2)CSc1nc2c(o1)cccc2	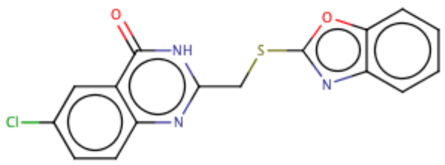	6.2928883
59	ZINC54581012	O=C(Nc1ccc(c(c1)Cl)Cl)Nc1ccc(cc1)N1CCCCC1	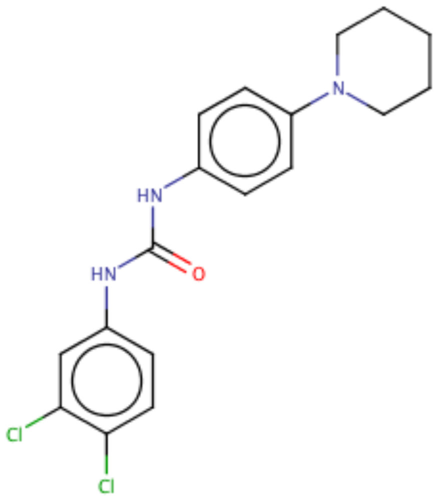	4.8949908
60	ZINC00924062	Clc1ccc(cc1)S(=O)(=O)N(Cc1cc2c(C)cc(cc2[nH]c1=O)C)Cc1ccc2c(c1)OCO2	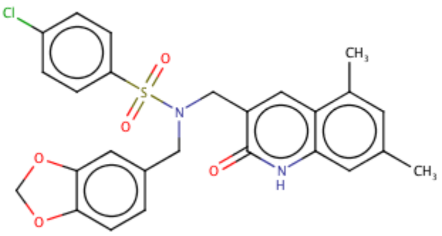	5.591317
61	ZINC17013891	Cc1nc(Nc2ccc(cc2)NS(=O)(=O)c2cccc(c2)Cl)nc(c1)N1CCCCC1	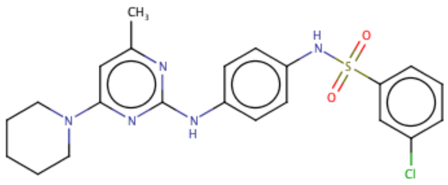	5.36896
62	ZINC10877144	CCn1c(=O)n(c2c1cccc2)Cc1nc(oc1C)c1cccs1	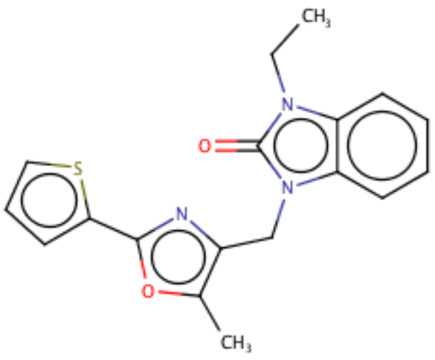	3.9105695

63	ZINC83537860	CCN(S(=O)(=O)c1ccc(c(c1)C(F)(F)F)C#N)c1cccnc1	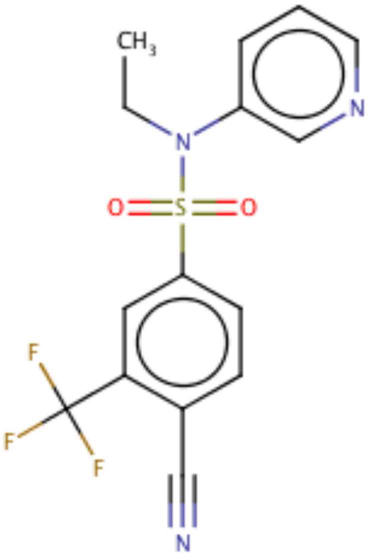	5.0422614
64	ZINC91636449	CSc1ncc(cn1)C(=O)N1CCC[C@@H]1c1onc(n1)c1ccccc1	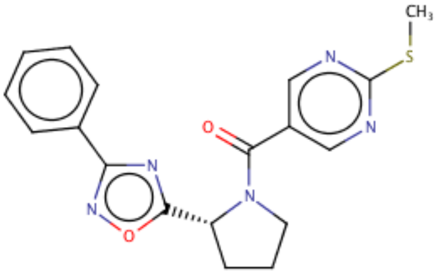	5.2808292
65	ZINC04776575	Fc1c(Oc2cccc3c2ncc3)c(F)c(c(c1F)C(F)(F)F)F	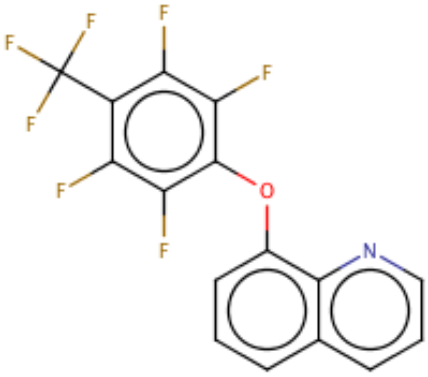	5.2331511

66	ZINC16971039	OC(c1ccccc1)(c1ccccc1)C[C@@H](c1cccc2c1cccc2)c1ccccc1	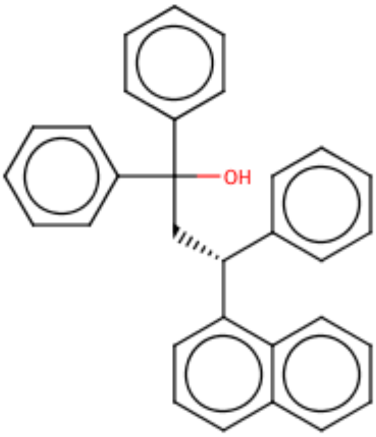	5.2560289
67	ZINC39391565	Clc1ccc2c(c1)[C@H]1Oc3cc(Cl)ccc3[C@@H]3N1[C@@H](O2)c1cc(Cl)ccc1O3	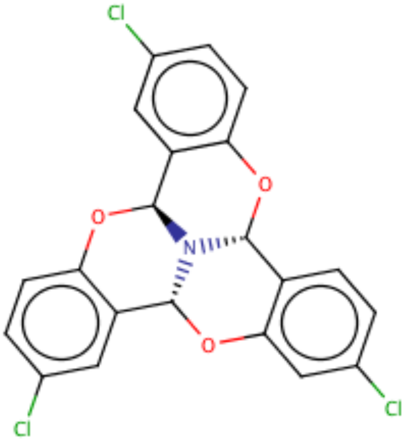	4.8347449

68	ZINC59047046	O=C(Nc1nnc(s1)SCc1ccc(cc1)Cl)CSc1nnc(n1N)CCc1ccccc1	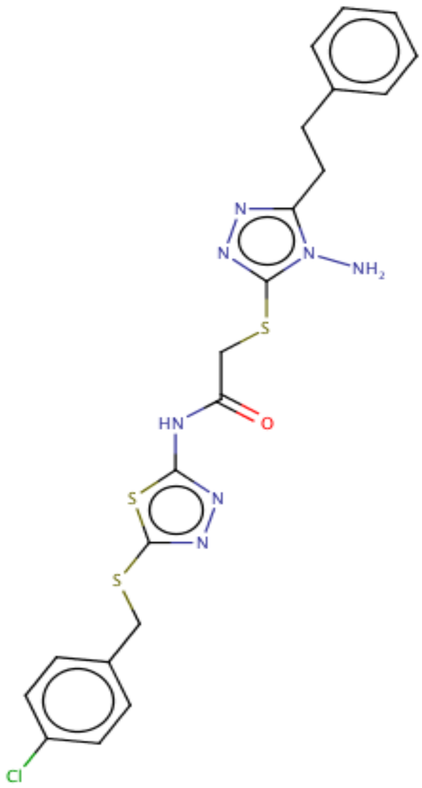	6.1241522
69	ZINC12625468	CCOC(=O)c1c(NC(=O)CSc2cc(C)c3c(n2)cc(cc3)OC)sc2c1CCCC2	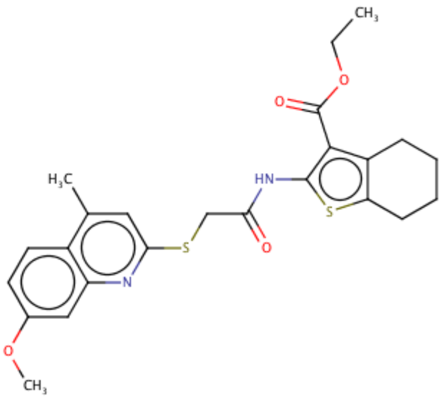	7.5485037

70	ZINC09610473	O=C(N1CCC(CC1)c1c(cnn1c1ccccc1)C(=O)N1CC[C@@H](C1)c1ccccc1)OC(C)(C)C	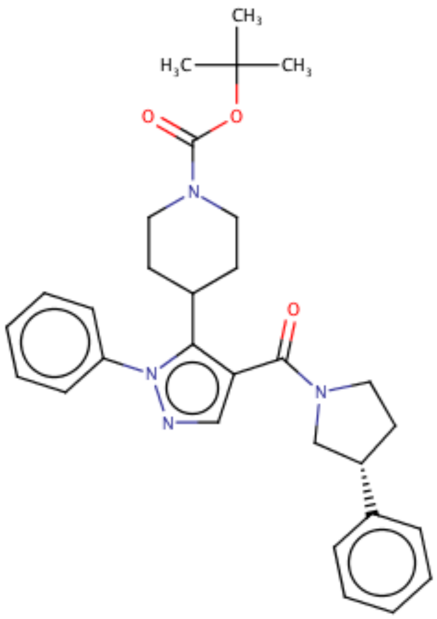	4.9914512
71	ZINC36691722	COc1cccc2c1nc(s2)N(C(=O)c1nccnc1)Cc1ccccn1	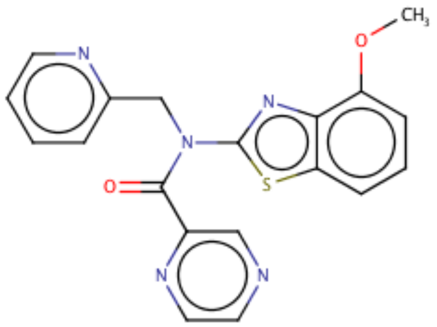	6.1128335
72	ZINC02398782	C=CCc1ccccc1OCCOCCn1c(nc2c(c1=O)cccc2)c1ccc(cc1)OC	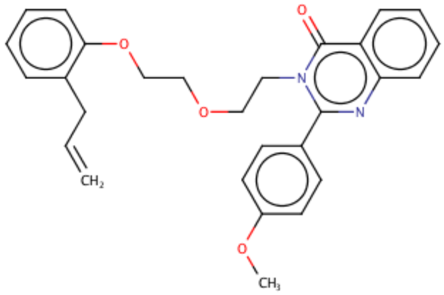	5.089154

73	ZINC12791027	COc1ccc(cc1c1scc(n1)CN1C(=O)c2c(S1(=O)=O)cccc2)Br	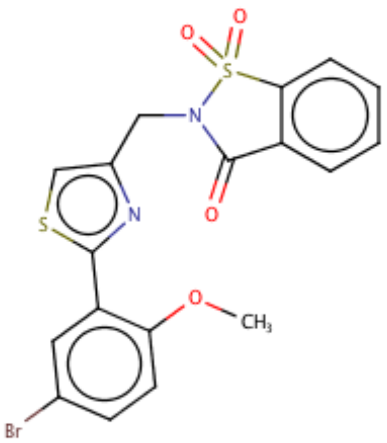	6.3387781
74	ZINC36074317	CCCN(C(=O)c1ccc2c(c1)OCO2)CC(=O)N1CCN(CC1)c1ccc(nn1)c1ccccc1Cl	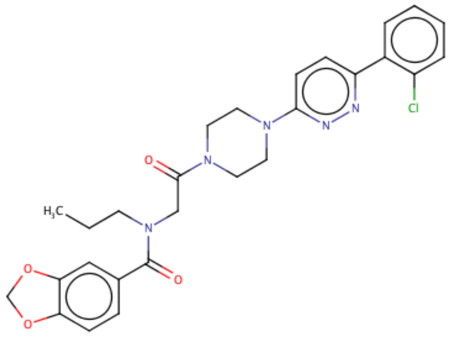	5.0062977
